# Zinc deficiency as possible link between immunosenescence and age-related diseases

**DOI:** 10.1186/s12979-025-00511-1

**Published:** 2025-05-19

**Authors:** Michael Tobias Schulz, Lothar Rink

**Affiliations:** Institute of Immunology, Faculty of Medicine, RWTH Aachen University Hospital, Pauwelsstraße 30, 52074 Aachen, Germany

**Keywords:** Ageing, Age-related Diseases, Immunosenescence, Inflammaging, Zinc

## Abstract

As global life expectancy increases, research reveals a critical challenge in aging: the progressive deterioration of immune function, termed immunosenescence. This age-related immune decline is characterized by a complex dysregulation of immune responses, which leaves older adults increasingly vulnerable to infections, chronic inflammatory states, and various degenerative diseases. Without intervention, immunosenescence significantly contributes to morbidity and mortality among the elderly, intensifying healthcare burdens and diminishing quality of life on both individual and societal levels. This review explores the essential role of zinc, a trace element critical for immune health, in mitigating the impact of immunosenescence and slowing the cascade of immunological dysfunctions associated with aging. By modulating the activity of key immune cells and pathways, zinc supplementation emerges as a promising approach to strengthen immunity, reduce oxidative stress, and counteract "inflammaging," a state of chronic, low-grade inflammation that accelerates tissue damage and drives disease progression. Zinc’s involvement in cellular defense and repair mechanisms across the immune system highlights its ability to enhance immune cell functionality, resilience, and adaptability, strengthening the body's resistance to infection and its ability to manage stressors that contribute to diseases of aging. Indeed, zinc has demonstrated potential to improve immune responses, decrease inflammation, and mitigate the risk of age-related conditions including diabetes, depression, cardiovascular disease, and vision loss. Given the prevalent barriers to adequate zinc intake among older adults, including dietary limitations, decreased absorption, and interactions with medications, this review underscores the urgent need to address zinc deficiency in aging populations. Recent findings on zinc’s cellular and molecular effects on immune health present zinc supplementation as a practical, accessible intervention for supporting healthier aging and improving quality of life. By integrating zinc into targeted strategies, public health efforts may not only sustain immunity in the elderly but also extend healthy longevity, reduce healthcare costs, and potentially mitigate the incidence and impact of chronic diseases that strain healthcare systems worldwide.

## Background

As the global population continues to age, the prevalence of age-related diseases such as cardiovascular disorders, neurodegenerative conditions, diabetes, and various types of cancer is expected to increase substantially. This demographic shift presents critical public health challenges, as older adults face not only the physical effects of these conditions but also an elevated vulnerability to infection and immune dysfunction [1]. A key factor in this vulnerability is the age-related decline in immune function, a phenomenon known as immunosenescence, which affects both innate and adaptive parts of the immune system. Immunosenescence results in a compromised immune response, impairing the body´s ability to defend itself against pathogens and adapt to new infections [2]. Compounding these effects is “inflammaging”, a state of chronic, low-grade inflammation that develops with age and further disrupts immune regulation. Inflammaging is associated with increased susceptibility to infections, autoimmune disorders and various age-related diseases, creating a feedback loop that accelerates the breakdown of immune health and increases morbidity and mortality risks among elderly [3, 4].

One of the essential elements in supporting immune health, particularly in the context of aging, is zinc, a trace element involved in numerous biological processes. Zinc plays a critical role in immune maintenance and regulation, as it influences DNA synthesis, cell division, and antioxidant defense mechanisms, functions vital to the integrity and effectiveness of immune responses. Zinc is necessary for the optimal function of key immune cells, including, neutrophils, macrophages, natural killer (NK) cells, and lymphocytes, all of which contribute to a robust and responsive immune system [5]. Among older adults, zinc deficiency is a common issue, arising from factors such as reduced dietary intake, decreased absorption efficiency, and altered metabolic requirements [6, 7]. This deficiency exacerbates immunosenescence and inflammaging, leading to increased inflammation, impaired immune defense, and a greater risk of chronic diseases.

The governmental German nutrition study conducted in 2008 revealed that 44% of men and 27% of women aged 65 to 80 years did not reach the recommended daily zinc intake [8]. Since then, dietary zinc intake recommendations have been revised upward. When the 2008 data are reanalyzed using the current guidelines established by the European Food Safety Authority (EFSA) [9], it is estimated that approximately 75% of individuals aged 65 to 80 years in Germany do not reach the recommended dietary zinc intake. These individuals are not definitively zinc deficient but are at greater risk for zinc deficiency or may have an inadequate zinc status, as zinc deficiency is defined by serum zinc levels below 70 µg/dL in fasting state [10]. Given this high prevalence of inadequate zinc status and likely also zinc deficiency with its adverse effect on immune function in aging populations [11], understanding and addressing zinc´s role in the immune system is critical to develop strategies that support healthier aging.

Recent research has shed light on the potential of zinc supplementation as a valuable approach to counteract immunosenescence and its associated health impacts. Zinc has been shown to modulate several crucial immune pathways, enhancing chemotaxis in neutrophils [12], promoting cytotoxic activity in NK cells [13], supporting macrophage phagocytic function [14], and preserving responsiveness of T and B cells [15, 16], which are essential components of adaptive immunity. Furthermore, zinc´s antioxidant properties contribute to its protective role against oxidative stress, a major driver of cellular aging and tissue damage in older adults [17]. Zinc also influences key gene expression pathways, including the nuclear factor kappa-light-chain-enhancer of activated B-cells (NF-κB) and peroxisome proliferator-activated receptor (PPAR) signaling, which are critical for managing inflammatory responses and enhancing cellular resilience. By targeting these pathways, zinc can potentially reduce systemic inflammation and oxidative damage, thereby supporting immune cell health and function across both innate and adaptive immunity [18, 19].

Beyond its cellular effects, zinc´s influence has broader potential in impacting the aging process and addressing various age-related diseases. By correcting cellular dysfunction often seen with aging, zinc supplementation may help delay the onset and progression of conditions such as age-related macular degeneration [20], diabetes mellitus [21], wound-healing impairments [22], depression [23] and cardiovascular disease [24], ultimately improving health outcomes for older adults. Zinc´s ability to reduce inflammation and enhance immune response makes it a promising agent for mitigating these chronic diseases, which are prevalent in aging populations and often worsen with immune decline. Its antioxidative and anti-inflammatory properties are essential for cellular protection and repair, aiding in the maintenance of tissue integrity and function across multiple organ systems as individuals age. In AMD, zinc may help to slow down retinal degeneration and preserve vision [20, 25]. In diabetes, it plays a role in insulin regulation and blood sugar control [21]. In wound healing it supports tissue regeneration and immune defense [22]. In depression it influences neurotransmitter balance and reduces inflammation [26, 27], and in cardiovascular disease, zinc helps to reduce oxidative stress and vascular inflammation [17, 28], which are central to preserving heart health in aging populations.

Unlike certain nutrients, such as fat-soluble vitamins or iron, which the body can store for extended periods, zinc lacks a dedicated storage system [29, 30]. This means the body depends on a steady dietary supply to maintain adequate levels for vital physiological functions [31]. This is especially concerning for vulnerable populations, such as older adults, who may face challenges in maintaining adequate zinc intake, as they may experience reduced appetite, changes in taste, dietary restrictions, poor dental status and age-related digestive issues that hinder nutrient absorptions [32–35]. Medications, such as penicillamine, tetracyclines, ACE inhibitors and angiotensin-2 receptor blockers, frequently prescribed in the aged population can also interfere with zinc absorption, further compounding the risk of deficiency. Thiazide diuretics increase urinary excretion of zinc [36–39].

Limited dietary variety and a decrease in overall nutrient intake can result in lower zinc levels among elderly individuals, creating a deficiency that has significant implications for their immune health and overall well-being. Addressing zinc deficiency through dietary improvements and targeted supplementation could therefore play a critical role in maintaining immune resistance and reducing the impact of immunosenescence.

**Fig. 1:**
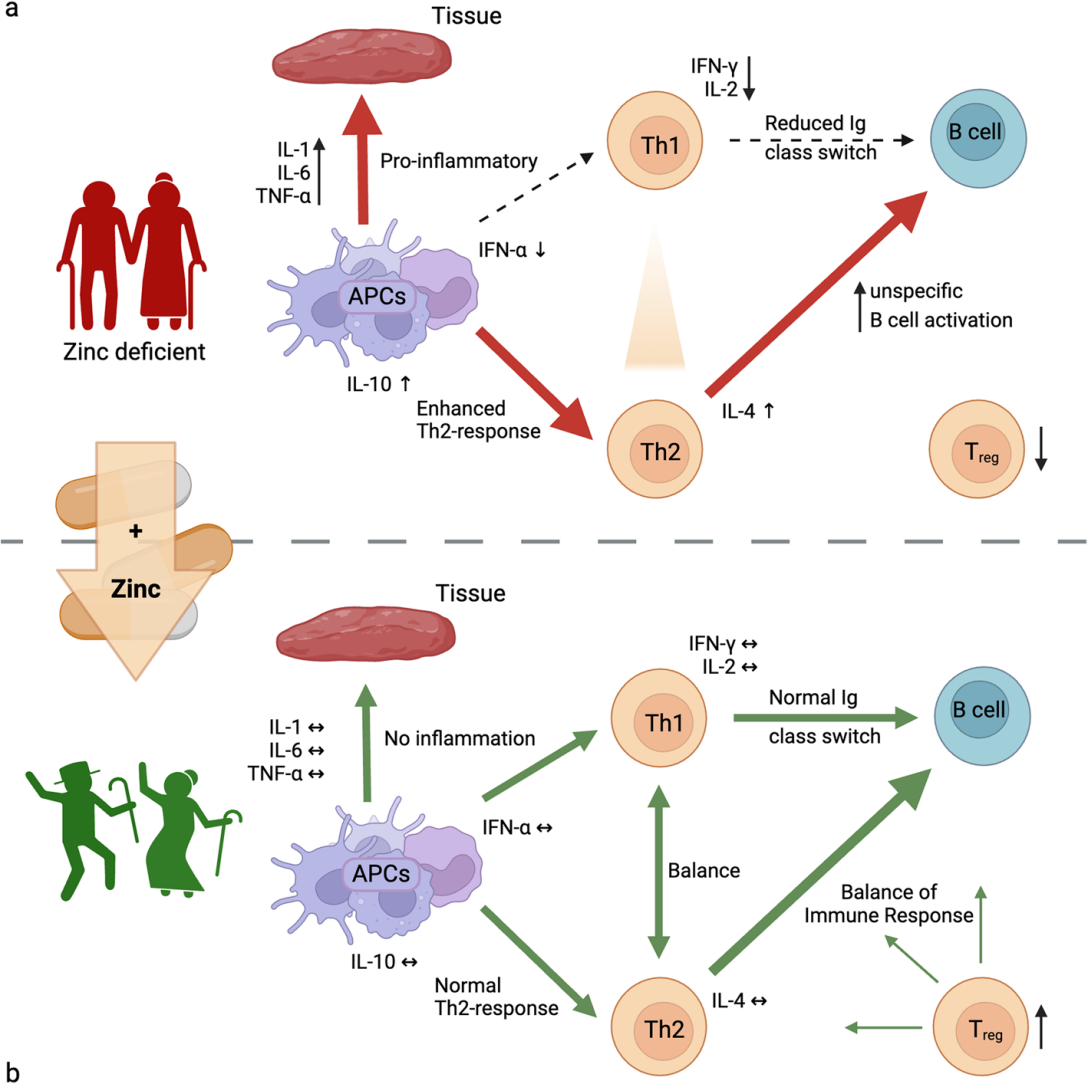
Overview of Zinc Status and Its Impact on Immune Function in Elderly Individuals  Dysregulated immune responses and unhealthy increased reactions associated with zinc deficiency,:  Restored immune balance and improved immune function achieved through zinc supplementation, (↑): increased, (↓): decreased, (↔): balanced (**a**) In aged and zinc deficient individuals, immune system dysregulation is characterized by increased pro-inflammatory cytokine production (IL-1, IL-6, TNF-α) by antigen-presenting cells, enhanced Th2 responses, and reduced Th1 responses. This leads to decreased IFN-γ and IL-2 production, reduced immunoglobulin (Ig) class switching, unspecific B cell activation, and decreased T regulatory cell function, promoting an inflammatory state and impaired immune balance. **b** Zinc supplementation restores immune homeostasis by reducing inflammation, balancing Th1/Th2 responses, and maintaining normal cytokine levels and B cell function. The presence of zinc supports APC function, enhances regulatory T cell​ activity, and promotes a balanced immune response in elderly individuals, mitigating immunosenescence. Created in https://BioRender.com

This review offers a comprehensive examination of the role of zinc in supporting immune homeostasis and countering the effect of immunosenescence. By exploring zinc´s influence on each component of the immune system, from innate responses mediated by neutrophils and macrophages to adaptive immunity regulated by T and B lymphocytes, this review underlines the importance of zinc for immune health in aging populations. Furthermore, the review delves into the clinical evidence supporting zinc supplementation as a strategy to decrease the severity of age-associated inflammatory conditions and chronic diseases, highlighting its potential to improve quality of life for older adults. By analyzing zinc´s molecular pathways, cellular interactions, and its broader impact on inflammation, this review aims to emphasize the significance of adequate zinc intake and illustrate how zinc supplementation could serve as a feasible, accessible, and effective approach to support immune health and enhance longevity among aging individuals (Fig. 1).

## Zinc and its key proteins

Zinc is an essential trace element that plays a critical role in numerous biological processes, functioning both as a structural and catalytic cofactor. It is indispensable for the activity of more than 300 enzymes [40] and is involved in a wide range of cellular functions, including protein synthesis, DNA repair, and cell division [41]. Its cellular regulation is tightly controlled by zinc transporters, particularly the Zip (Zrt/Irt-like protein) family (Zip1-14/SLC39 A1-14), which facilitates zinc influx into the cytoplasm, and the ZnT (Zinc Transporter) family (ZnT1-10/SLC30 A1-10), which mediates zinc efflux to maintain homeostasis. Among the Zip family, ZIP4 is especially critical, as it is responsible for dietary zinc uptake in the intestine [42]. Mutations in Zip4 lead to acrodermatitis enteropathica, a rare but severe zinc deficiency disorder [43]. Zip7 is critical for intracellular zinc release from the endoplasmic reticulum into the cytosol and is essential for proper B-cell receptor signaling. A mutation in Zip7 has been identified as the cause of an autosomal recessive disorder, which is characterized by the lack of B cells and immunoglobulins, resulting in early-onset infections [44]. Other important ZIPs include Zip8, which is involved in T cell activation and IFN-γ expression [45], and Zip14, which plays a role in zinc mobilization during infection and acute-phase responses [46].

In contrast, the ZnT family primarily functions to export zinc out of the cytoplasm or into organelles. ZnT1 is the most well-known member, as it is responsible for reducing intracellular zinc levels by exporting it across the plasma membrane [42]. ZnT2 is critical for zinc secretion into breast milk [47], while ZnT3 is essential in neurons for packaging zinc into synaptic vesicles, a process vital for neurotransmission and brain function [27].

Metallothioneins (MTs), small cysteine-rich proteins, act as zinc-binding proteins to buffer and store zinc ions, protecting cells from oxidative stress and regulating zinc availability [48]. Zinc predominantly functions as a cofactor in enzymes, stabilizing protein structures through its interaction with specific amino acid residues, such as cysteine and histidine, thereby contributing to the secondary and tertiary of target proteins [49, 50]. It is essential in forming zinc finger motifs, which are critical for DNA binding and transcriptional regulation. Additionally, zinc ions can regulate enzymatic activity, DNA binding, and protein–protein interactions by inducing conformational changes in target proteins [49]. Unlike many cofactors that directly participate in redox reactions, zinc's biological role largely involves structural stabilization and catalytic activity, making it indispensable in processes such as transcription, signal transduction, immune function, and apoptosis [5] (Tables 1 and 2).

**Table 1:** Zinc transporters and their immunological relevance

Protein	Distribution	Immunological relevance	References
ZnT1	Ubiquitous	-	[51]
ZnT2	Widely expressed	Mutations lead to low zinc in maternal milk and thereby neonatal zinc deficiency (humans only)	[52, 53]
ZnT3	Brain	-	[54, 55]
ZnT4	Ubiquitous	Lethal milk mutant leads to less zinc in milk and neonatal zinc deficiency (mice only, human ZnT2)	[56]
ZnT5	Ubiquitous	Loss of function leads to impaired mast cell function	[57]
ZnT6	Widely expressed	-	
ZnT7	Widely expressed	-	[58, 59]
ZnT8	Pancreas	Autoantigen in type I diabetes mellitus	[60]
ZnT9	Brain, muscle kidney	-	[61]
ZnT10	Small intestine, liver, brain	-	[62, 63]

Modified from Hara et al. 2017 [64]

**Table 2:** Zip proteins and their immunological relevance

Protein	Distribution	Immunological relevance	References
Zip1	Ubiquitous	-	[65]
Zip2	Liver, ovary, skin, dendritic cell	-	[66]
Zip3	Widely expressed	Knockout leads to impaired T cell development	[67]
Zip4	Small intestine	Mutation leads to acrodermatitis enteropathica	[43]
Zip5	Small intestine, kidney, pancreas	-	[68]
Zip6	Widely expressed	Necessary for T cell activation	[69, 70]
Zip7	Widely distributed	Necessary for B cell development	[44]
Zip8	Widely expressed	Knockout leads to decreased T cell activation and IFN-γ production, Missense mutation is linked to Crohn´s disease	[45, 71]
Zip9	Widely expressed	-	[72]
Zip10	Widely expressed	Regulates intensity of B-cell receptor signaling, Essential for macrophage survival during inflammatory events	[73, 74]
Zip11	Brain, gastrointestinal tract, stomach, cecum, colon, mammary gland, testis	-	[75–78]
Zip12	Brain, lung, smooth muscle testis	-	[79–81]
Zip13	Hard and connective tissues	-	[82, 83]
Zip14	Widely expressed	Deletion results in the reduction of MHC-II on small intestinal epithelial cells; induced by proinflammatory signals	[84–88]

Modified from Hara et al. 2017 [64]

## Recommended zinc intake

Various international organizations, including the European Food Safety Authority (EFSA), the German Nutrition Society (DGE), the National Institutes of Health (NIH) and the World Health Organization (WHO), provide guidelines for zinc intake to support optimal health (Table 3).

**Table 3:** Comparison of zinc intake recommendations among different health organizations

	**Bioavailability**	**RNI (mg/day)**		**UL**
		**male**	**female**	
**EFSA** [9]	Phytate intake per day:			
	300 mg	9.4 mg	7.5 mg	25 mg
	600 mg	11.7 mg	9.3 mg	
	900 mg	14.0 mg	11.0 mg	
	1200 mg	16.3 mg	12.7 mg	
**DGE** [89]	Phytate intake per day:			
	330 mg	11 mg	7 mg	25 mg
	660 mg	14 mg	8 mg	
	990 mg	16 mg	10 mg	
**WHO** [90]	High bioavailability^a^	4.2 mg	3.0 mg	45 mg^b^
	Moderate bioavailability^a^	7.0 mg	4.9 mg	
	Low bioavailability^a^	14.0 mg	9.8 mg	
**NIH** [91]	Independent from bioavailability	11 mg	8 mg	40 mg

*RNI* Recommended nutrient intake, *UL* Tolerable Upper Intake Level, *EFSA* European Food Safety Authority, *DGE *German Nutrition Society, *WHO* World Health Organization, National Institutes of Health

^a^Low availability: phytate–zinc molar ratio > 15, moderate availability: phytate–zinc molar ratio between 5 and 15, high availability: phytate–zinc ratio < 5

^b^ for men

EFSA provides zinc intake recommendations that account for dietary phytate levels. Phytates are naturally occurring compounds found in plant-based foods like grains, legumes, and seeds that bind to zinc in the digestive tract, reducing its absorption and bioavailability. For men, the recommended intake ranges from 9.4 mg per day with low phytate levels (300 mg/day) to 16.3 mg per day with high phytate intake (1200 mg/day). For women, the recommendations range from 7.5 mg to 12.7 mg per day across the same phytate levels [9]. Similarly, DGE suggests zinc intakes of 11 mg per day for men and 7 mg per day for women when phytate levels are low (330 mg/day). At higher phytate levels (990 mg/day), these recommendations rise to 16 mg per day for men and 10 mg per day for women [89].

WHO bases its zinc recommendations on bioavailability. In diets with high zinc bioavailability, men require 4.2 mg per day and women 3.0 mg per day. For moderate bioavailability, the recommended intake increases to 7.0 mg per day for men and 4.9 mg per day for women. Low bioavailability diets necessitate the highest zinc intakes: 14.0 mg per day for men and 9.8 mg per day for women [90]. Meanwhile, NIH recommend zinc intakes that are independent of phytate levels, suggesting 11 mg per day for men and 8 mg per day for women [91].

Excessive zinc intake, however, can lead to toxicity and significant health complications. Symptoms of acute zinc toxicity include gastrointestinal distress, such as nausea, vomiting, abdominal cramps, and diarrhea [92, 93]. Chronic high zinc consumption poses more serious risks, such as immune dysfunction and interference with copper metabolism. Zinc competes with copper for absorption in the gastrointestinal tract, and prolonged excessive zinc intake can lead to copper deficiency. This condition may result in anemia, neutropenia, and neurological symptoms such as ataxia and peripheral neuropathy. Other symptoms of zinc toxicity include headaches, fatigue, and disruptions in lipid metabolism [92–94].

To minimize these risks, tolerable upper intake levels (ULs) for zinc have been established by various organizations. EFSA and DGE set the UL at 25 mg per day for adults [9], while the NIH recommends a higher UL of 40 mg per day [91]. However, the upper limit of 40 mg is likely too high, as studies indicate that negative side effects can occur below this threshold. A limit of 25 mg may be a more appropriate upper boundary for zinc intake [92].

To assess whether dietary zinc intake is sufficient or excessive, the use of a food questionnaire can be particularly valuable. This tool helps to estimate zinc intake by evaluating the frequency and quantity of zinc-rich or phytate-rich foods in the diet, providing an effective method for determining whether dietary adjustments are necessary to meet individual nutritional needs [95]. Furthermore, an application accessible at www.zink-app.de enables individuals to assess their individual zinc status [96].

## Sex- and gender-related differences in immunity

Immunosenescence differs significantly between sexes and genders, influenced by biological, hormonal, and environmental factors. Women generally exhibit stronger immune responses than men throughout much of their lives, a phenomenon attributed to differences in sex hormones and genetic factors [97]. Estrogen receptors are present on many immune cells, including neutrophils, macrophages, dendritic cells, natural killer cells, T lymphocytes, and B lymphocytes [98]. Estrogen, a key hormone in females, plays a critical role in enhancing immune function. It stimulates the production of immunoglobulin (Ig) G and IgM antibodies [99], whereas testosterone has the opposite effect, reducing the production of these immunoglobulins [100]. Women also have higher numbers of CD4^+^ T cells [101], and estrogen increases the expression of interferon (IFN)-γ, an important cytokine for immune activation [102, 103].

The phagocytic activity of neutrophils, macrophages, and other antigen-presenting cells is more pronounced in females than in males [104]. Additionally, chronic estradiol application promotes the expression of interleukin (IL)−1β, IL-6, IL-12p40, and Inducible nitric oxide synthase (iNOS) in macrophages following lipopolysaccharide (LPS) stimulation, further enhancing immune responses [105]. Women also produce significantly more IFN-α after Toll-like receptor (TLR) 7 activation compared to men [106, 107]. These factors collectively provide women with a robust capacity to combat infections and mount stronger immune responses.

The genetic advantage conferred by the presence of two X chromosomes in females further contributes to their immunological resilience. While the X chromosome does carry numerous immune-related genes, the biological implications are more nuanced than simple redundancy. To balance gene expression differences, one of the two X chromosomes in female cells undergoes X chromosome inactivation (XCI), a process that epigenetically silences most genes on the inactive X chromosome (Xi). Approximately 15%–30% of Xi-linked genes avoid this silencing, with patterns differing across cellular context, genetic background and developmental stages [108, 109]. This incomplete inactivation mosaicism results in a partially higher gene expression and provides a functional diversity that can enhance immune responses and compensate deleterious mutations. In contrast, males, with only one X chromosome, lack both the protective effects of mosaicism and the potential benefits of increased gene expression conferred by XCI escape. This may contribute to sex-based differences in immune function and disease susceptibility, while in females, the same mechanism may lead to heightened immune responses and an increased risk of autoimmunity [98, 110].

However, this sex-based advantage diminishes with age, particularly after menopause. The menopausal transition involves a significant decline in estrogen levels, which negatively impacts the immune system [111]. The protective effects of estrogen wane, leading to a reduction in immune cell populations. Postmenopausal women exhibit lower numbers of B lymphocytes and CD4^+^ T lymphocytes [112]. Chronic increases in inflammatory markers, including IL-1β, IL-6, and IL-10, are observed in serum during and after menopause [113–115]. Although IFN-γ levels initially rise during menopause, they eventually decrease to levels lower than those observed before menopause [113]. A comparison of the gene expression profiles of peripheral blood mononuclear cells (PBMCs) in elderly men and women with younger controls revealed that 48 signaling pathways are altered in females, compared to only 29 in males. This finding suggests that the aging immune system undergoes more extensive changes in women, particularly in T cell protection, which weakens, while inflammation increases [116].

This hormonal shift largely explains the narrowing of the immunological gap between postmenopausal women and men of the same age. Before menopause, men are more at risk for infections such as hepatitis, meningococcal disease, and pneumococcal disease due to their comparatively weaker immune responses. However, as women age and their immunological advantage diminishes and their risk for these infections increases to levels similar to those observed in men [117]. Hormone replacement therapy has been shown to mitigate some of these immunological alterations. For example, it increases the number of B cells [118] and decreases chronically elevated pro-inflammatory cytokines, such as IL-6, through estrogen supplementation [119].

Gender also intersects with environmental and social factors that influence aging and immune function. Societal norms and behaviors may lead to differences in lifestyle, access to healthcare, and exposure to chronic stress, all of which impact the immune system over time [120]. Furthermore, non-binary and transgender individuals may experience unique interactions between hormonal therapies, environmental factors, and aging. However, this area remains under-researched, highlighting the need for further studies.

In summary, while women generally possess stronger immune function than men due to hormonal and genetic advantages, this disparity diminishes after menopause as estrogen levels decline. Since this review focuses on the immunological changes in elderly individuals, particularly postmenopausal populations, further exploration of sex differences will not be addressed in detail.

## Changes in innate immunity

### Granulocytes

**Fig. 2:**
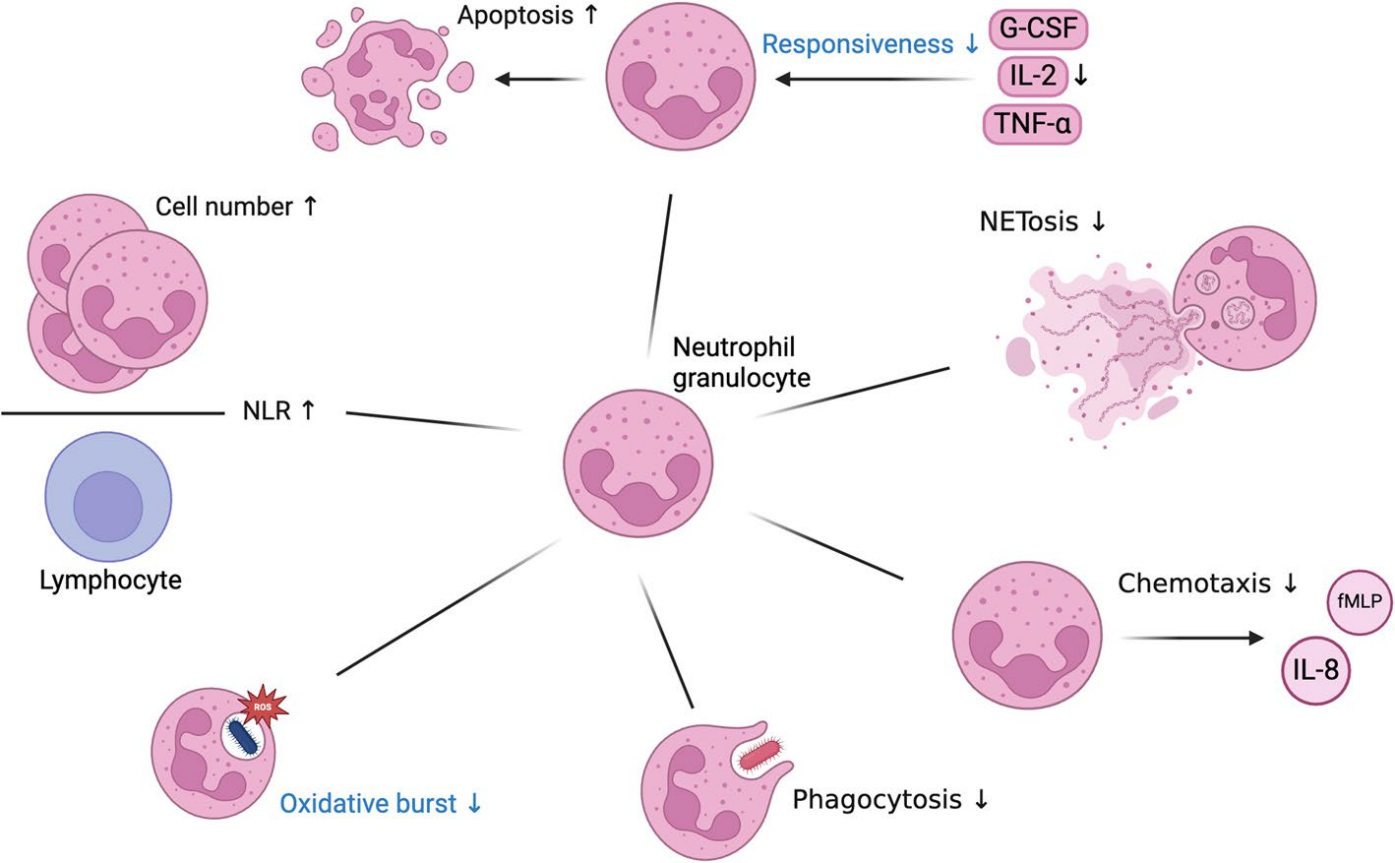
Functional Decline of Neutrophils in Aging and Zinc Deficiency.  : Alterations due to aging, but not described in zinc deficiency, (Black) Alterations described in both, zinc deficiency and aging, (↑): increased, (↓): decreased Created in https://BioRender.com

Granulocytes, which include neutrophils, eosinophils, and basophils, play essential roles in the innate immune response. These cells primarily perform three key functions: phagocytosis, degranulation and the production of cytokines and chemokines [121]. However, age-related changes in neutrophil function have significant implications for the immune response (Fig. 2).

Neutrophil function is thought to be compromised in older adults, as indicated by increased morbidity and death caused by bacterial infections with advancing age [122]. Interestingly, while the total number of neutrophils increases during ageing [123], evidence suggests that their functional capacity declines. Changes in the expansion of polymorphonuclear precursors to granulocyte-colony stimulating factor (G-CSF) have been observed in elderly individuals [124], although responses to other stimulants like IL-3 and granulocyte–macrophage colony stimulating factor (GM-CSF) remain unchanged, ensuring sufficient neutrophilia during infection [125, 126].

The neutrophil-to-lymphocyte ratio (NLR) increases during aging. It has been recognized as a prognostic biomarker for assessing the progression of neoplastic diseases, predicting the onset of chronic degenerative conditions, and serving as a potential indirect indicator of healthy aging. Notably, the NLR typically increases in association with neoplastic diseases, reflecting systemic inflammation, while a decrease in NLR may be indicative of healthy aging [123, 127, 128]. Consistent with its role in modulating immune function, plasma zinc is inversely associated with the neutrophil-to-lymphocyte ratio [129]. This suggests that zinc supplementation in the elderly may help restore a healthier neutrophil-to-lymphocyte ratio, potentially improving immune function during aging.

The functional decline in neutrophils is associated with several factors, leading to impaired apoptosis regulation. Older neutrophils are at a higher risk of apoptosis due to a lower responsiveness to factors such as G-CSF, IL-2, or tumor necrosis factor (TNF)-α that would normally prevent them from undergoing programmed cell death [130, 131]. This phenomenon may be linked to an altered ratio in anti-apoptotic and pro-apoptotic proteins. For example, the Mcl-1/Bax ratio increases following GM-CSF stimulation in neutrophils from individuals of a younger age, whereas this ratio remains unchanged in neutrophils from older individuals [132, 133]. Zinc supplementation, of patients with down syndrome, which often goes along with zinc deficiency, could reduce the number of apoptotic cells [134, 135]. There is a significant age-related decline in oxidative burst activity, particularly in older adults. In nonagenarians, there is a higher expression of macrophage-1 antigen (MAC-1), which is involved in neutrophil adhesion and migration, compared to younger and middle-aged individuals. Interestingly, neutrophil zinc content is higher in both nonagenarians and young adults compared to middle-aged individuals, suggesting a potential age-related redistribution of zinc. Additionally nonagenarians exhibit increased superoxide dismutase (SOD) activity, which may represent a compensatory mechanism against oxidative stress [136].

Zinc influences various neutrophil functions, including migration, chemotaxis, and the oxidative burst. Zinc deficiency impairs neutrophil chemotaxis, as shown in models of crush syndrome where zinc chelation reduced neutrophil infiltration and muscle injury, indicating that adequate zinc levels are crucial for optimal neutrophil function migration [137]. Zinc deficiency has also been linked to inflammatory diseases, including acute lung injury (ALI), characterized by excessive neutrophil recruitment and hyperactivation. However, administering zinc to mice 24 h before LPS-induced ALI significantly reduced neutrophil recruitment to the lungs, inhibited their hyperactivation, and consequently decreased lung damage [138].

In zinc-treated hemodialysis patients, granulocytes exhibit enhanced responsiveness to stimuli like zymosan-activated serum, showing greater chemokinetic activity and motility [139]. Zinc also promotes chemotaxis by increasing the expression of IL-8 [12, 140]. This could be due to a Zn-induced inhibition of phosphatase activity, leading to Mitogen-activated protein kinase (MAPK) and activator protein-1 (AP-1) activation, resulting in the enhanced IL-8 expression [140]. Additionally, zinc influences GM-CSF receptor expression on immune cells differently, increasing it on neutrophils but decreasing it on monocytes [141].

Neutrophils are critical first responders to infection or injury, and their ability to migrate toward chemotactic signals is essential for effective immune defense. Zinc, along with other trace elements like copper and nickel, enhances neutrophil chemotaxis, promoting their migration to sites of infection in a manner similar to the chemotactic factor N-formylmethionyl-leucyl-phenylalanine (fMLP). Zinc and nickel, in particular, double neutrophil migration towards fMLP, demonstrating a synergistic effect in enhancing chemotaxis [142]. Another study shows that chelation of zinc with the membrane permeable chelator *N*,*N*,*N′*,*N′*-tetrakis(2-pyridinylmethyl)−1,2-ethanediamine (TPEN) reduces granulocyte migration towards fMLP and IL-8 as well as phagocytosis, oxidative burst and granule release. But the direct action of zinc as chemoattractant, as previously mentioned [142], was not observed. TPEN also inhibited the secretion of both chemotactic IL-8 and anti-inflammatory cytokine IL-1ra in response to LPS stimulation [12]. Consistent with these findings that zinc deficiency impairs neutrophil chemotaxis, it has been observed that chemotaxis is diminished in elderly individuals as well [143–147].

After they arrived at their destination, polymorphonuclear neutrophils (PMN) have various options for neutralizing pathogens. The main ones are: Intracellular killing through oxidative burst, phagocytosis and degranulation. Most of them are constrained during zinc deficiency [12, 142]. Given that zinc deficiency is more prevalent in the elderly, ensuring adequate zinc intake may be particularly important for maintaining neutrophil function and reducing susceptibility to infections in older adults.

While it is established that phagocytosis is significantly impaired in the context of elderly individuals [147–149] as well as zinc deficiency [12], the impact of zinc on oxidative burst remains controversial. Zinc can both inhibit and enhance neutrophil respiratory burst, depending on the context. For instance, zinc inhibits oxidative burst in isolated neutrophils but enhances it in whole blood, suggesting that the cellular environment may influence zinc´s effect [150]. Moreover, zinc inhibits superoxide generation in peritoneal neutrophils at concentrations as low as 10 µM [151], while other studies report enhanced superoxide release and hydrogen peroxide production at higher zinc concentrations [152]. Zinc hydroxide has been found to induce both oxidative burst and phagocytosis in rat neutrophils, further highlighting its dual role in modulating immune responses [153]. However, in this study, zinc treatment in PMNs did not improve phagocytosis or bacterial killing *of Staphylococcus aureus* and *Staphylococcus epidermidis*, but it did improve cell membrane protection, potentially mitigating damage during immune responses [154]. In the context of aging, it has become increasingly evident that oxidative burst activity declines with age [136, 148, 155, 156]. However, this phenomenon appears to be context-dependent, as oxidative burst tends to decrease in suspended cells while exhibiting an increase in adhesive cells [157].

Zinc may influence neutrophil degranulation and oxidative metabolism by activating protein kinase C (PKC) and promoting myeloperoxidase (MPO) degranulation, leading to the production of reactive oxygen species (ROS), particularly hypochlorous acid, a potent antimicrobial compound [158, 159]. More recent studies show that zinc, at low concentrations (5–12.5 µM), activates NADPH oxidase via PKC, leading to superoxide radical production. Higher zinc concentrations facilitate the conversion of superoxide to hydrogen peroxide and HOCl through MPO, enhancing microbial killing [160].

Zinc´s role in NETosis, the process by which neutrophils release extracellular traps (NETs) composed of DNA, chromatin, and granule proteins to trap pathogens, is equally complex. PKC-mediated NET formation depends on ROS production by NADP oxidase. Zinc signaling is essential for NETosis, as zinc chelation by TPEN inhibits this process without affecting PKC activity or ROS production, suggesting zinc acts downstream of ROS [161]. Zinc supplementation increases NET release, although it does not alter NET efficacy in bacterial killing [162]. However, other studies show that zinc can inhibit NET release and degranulation, possibly by reducing citrullinated histone H3 levels [163]. The impact of aging on NETosis is not well studied yet. Some studies suggest reduced NET formation in neutrophils from older individuals [164, 165], while other ones indicate an increase in NET production with age [166]. However, this heightened production may be associated with diminished functionality and efficiency, likely due to alterations in NET composition and impaired clearance mechanisms [166].

Hence, maintaining adequate zinc levels is vital for supporting neutrophil function in the elderly, as these cells play a key role in the immune defense. Zinc enhances neutrophil activities such as migration, oxidative burst, and cytokine release, which tend to decline with age (Table 4).

**Table 4:** Comparison of granulocyte counts and functions in aged individuals and zinc deficient state

Cell type	Characteristics	Alteration		References	
		**Elderly**	**ZD**	**Elderly**	**ZD**
**Neutrophil granulocytes**					
	Cell Number	↗	↗	[123, 167, 168] ↑ [130, 169, 170] ↔	[171] ↔ [172, 173] ↑
	NLR	↑	↑	[123] ↑	[129] ↑
	Apoptosis	↑	↗	[130, 131] ↑	[136] ↘ [134, 135] ↑
	Oxidative Burst	↓	↔	[136, 148, 155–157] ↓	[150] ↓↑ [151–153] ↓
	Chemotaxis	↓	↓	[143–147] ↓	[12, 137, 139, 142] ↓
	Phagocytosis	↓	↓	[147–149] ↓	[12] ↓ [163] ↘
	NETosis	↓	↓	[164–166] ↓	[161–163, 174] ↓
**Basophil granulocytes**					
	Cell Number	↓	↑	[175] ↓	[172, 173] ↑
	Cell Number (tissue resident)	↑	-	[176] ↑	-
**Eosinophil granulocytes**					
	Cell Number	↓	↗	[168] ↓	[177] ↓ [172, 173, 178] ↑
	Cell number (tissue resident)	↔	↑	[168, 179] ↔	[180] ↑

*ZD* Zinc deficiency, ↓: decreased significantly, ↘: probably decreased; ↔ : no effect, or effect unclear, -: no data available, ↗: probably increased; ↑: increased significantly

### Mast cells

Mast cells are known to be central to various pathological processes that contribute to allergic reactions. Conditions such as anaphylaxis, atopic eczema, and asthma are among the disorders where their role is particularly significant [181].

The total number of mast cells has been found to increase with age [182, 183], a pattern that bears similarities to the response observed in zinc deficiency [184]. However, with advancing age, mast cells tend to enter a state of nonspecific preactivation, while their functional capacity upon stimulation becomes diminished [183]. This decline is reflected in the reduced frequency of degranulation observed in mast cells of aged skin [185].

Zinc is a necessary component for both degranulation as well as cytokine production in mast cells. The zinc chelator TPEN has been demonstrated to inhibit cytokine production, the secretion of lipid mediators and the release of histamine in mast cells. Conversely, zinc supplementation has been shown to reverse these inhibitory effects. Other metal chelators do not exert any influence on mast cell function [186]. Furthermore, mast cells express various zinc transporters, including ZnT4, which facilitate the uptake and storage of zinc. This is particularly important in the context of inflammation, where mast cells can rapidly regain high levels of labile zinc after degranulation [187]. In addition, findings demonstrate that ZnT5 is necessary for the development of contact hypersensitivity and mast cell-mediated delayed-type allergic responses, though it is not involved in immediate passive cutaneous anaphylaxis [57]. Zinc also alters the cytokine production of mast cells. L-type calcium channels mediate zinc signaling in mast cells, influencing the production of proinflammatory cytokines such as IL-6 in response to various stimuli, including LPS and IL-33 [188], (Table 5).

**Table 5:** Comparison of mast cell counts and functions in aged individuals and zinc deficient state

Cell type	Characteristics	Alteration		References	
		**Elderly**	**ZD**	**Elderly**	**ZD**
**Mast cells**					
	Cell number	↑	↗	[182, 189, 190] ↑	[184, 191] ↗
	Activation/Degranulation	↓	↓	[185] ↓	[186] ↓

*ZD* Zinc deficiency, ↓: decreased significantly, ↘: probably decreased; ↔ : no effect, or effect unclear; -, no data available, ↗: probably increased, ↑: increased significantly

### Natural killer cells

Aging significantly affects the number, function, and phenotype of NK cells, which are critical components of the innate immune system. While no changes in NK progenitor numbers have been observed in the peripheral blood or bone marrow [192], most studies report an increase in the total number of NK cells with age [193–195]. However, this increase is accompanied by a significant shift in NK cell subsets. There is a notable decrease in the percentage of CD56^bright^ cells, which are primarily responsible for cytokine secretion, including IFN-γ and TNF-α [195–197]. Conversely, there is an increase in CD56^dim^ cells, which are predominantly cytotoxic [196–198].

Despite the increased number of cytotoxic CD56^dim^ cells, age-related declines in NK cell cytotoxicity have been reported. These include impaired IFN-γ secretion, reduced expression of perforin and granzyme, and defective perforin release through degranulation [199–201]. Consistent with these findings, in vitro zinc chelation has also been shown to decrease NK cell cytotoxicity, which could be increased through zinc supplementation [202].

The ability of NK cells to recognize and kill target cells is also affected by changes in receptor expression**.** Aging results in reduced expression of activating receptors like NKp30 and NKp46, while the expression of inhibitory receptors like KLRG1 and killer cell immunoglobulin-like receptors (KIRs) increases [198, 201, 203]. Human leucocyte antigen (HLA)-C-specific KIRs can bind zinc, and chelating zinc reverses their inhibitory function, suggesting that zinc is essential for their activity. Zinc induces KIR-self-association, leading to the formation of multimers, a phenomenon not observed with other divalent cations [204–207]. Zinc supplementation possibly helps restoring NK cell functionality in elderly by reversing the inhibitory effects of KIRs, promoting effective target recognition, and enhancing the immune response.

Zinc also plays a pivotal role in enhancing NK cell function. For instance, peptidoglycan monomer zinc significantly improves cytotoxicity against NK-sensitive targets [208]. Studies show a positive correlation between NK cell numbers and vitamin D levels, while lifestyle factors like smoking and alcohol consumption negatively impact NK cell activity [209].

In elderly individuals, three months of zinc supplementation could temporarily boost NK cell activity [13]. Aging is associated with higher expression of metallothionein mRNA, reducing zinc bioavailability, and supplementation can restore zinc levels, enhancing NK cell cytotoxicity [210, 211].

Zinc also affects NK cell cytokine production. After stimulation with phytohemagglutinin (PHA), zinc promotes IFN-γ release and suppresses IL-10 release, shifting the immune response towards a Th1 phenotype. This cytokine shift, which enhances antiviral and antitumor immune responses, which are critical for the elderly to fight age-associated diseases, is linked to an increase in NK cell numbers, potentially explaining the enhanced IFN-γ production [212].

**Fig. 3:**
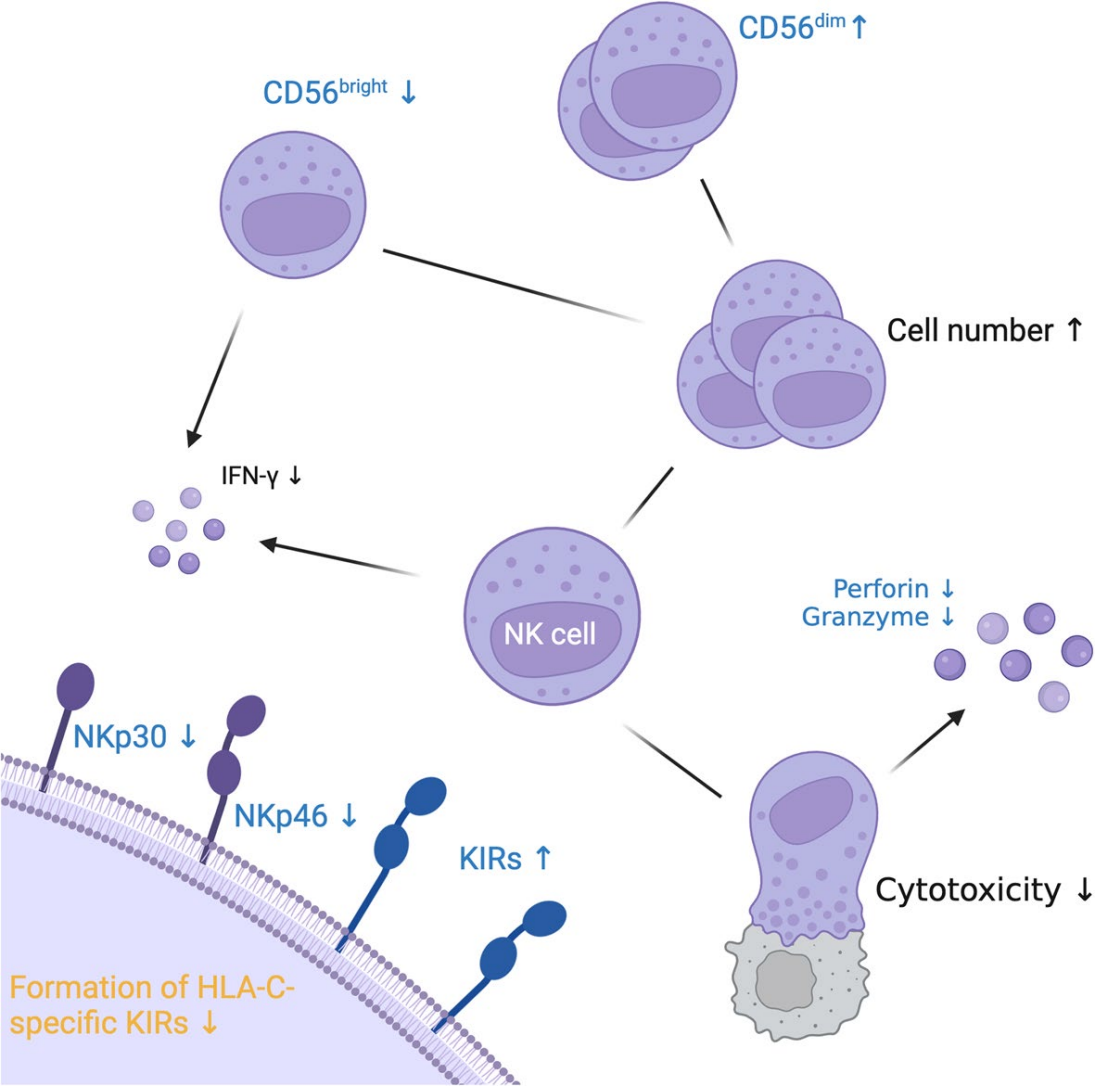
Alterations in Natural Killer Cell Function Associated with Aging and Zinc Deficiency.  : Alterations due to aging, but not described in zinc deficiency,  : Alterations due to zinc deficiency, but not described in aging, (Black) Alterations described in both, zinc deficiency and aging, (↑): increased, (↓): decreased Created in https://BioRender.com

Zinc´s role in NK cell development is further highlighted by its effects on CD34^+^ progenitor cells. When cultured from both young and elderly donors, CD34^+^ cells from young individuals predominantly developed into CD56^+^CD16^—^NK cells, while cells from elderly individuals exhibited a CD56^−^CD16^+^ phenotype with lower cytotoxic activity. Zinc supplementation increased the number and cytotoxic activity of NK cells in both age groups, and notably, zinc induced the expression of the transcription factor GATA-3, which is essential for NK cell maturation [192]. These findings underline zinc´s importance for both the development and functional regulation of NK cells across the lifespan, which could support aging immune systems against infections and cancers (Fig. 3 and Table 6).

**Table 6:** Comparison of NK cell counts and functions in aged individuals and zinc deficient state

Cell type	Characteristics	Alteration		References	
		**Elderly**	**ZD**	**Elderly**	**ZD**
**NK cells**					
	Cell number	↑	↗	[195, 201, 213, 214] ↑ [215] ↗	[172] ↔ [216] ↑
	CD56^bright^ cells	↓	-	[195–197] ↓	**-**
	CD56^dim^ cells	↑	-	[196–198] ↑	-
	Cytotoxicity	↓	↓	[199–201] ↓	[13, 202, 208] ↓
	KIR expression/formation	↑	↓	KIR expression: [198, 201, 203] ↑	Formation of KIR multimers: [204–207] ↓

ZD: Zinc deficiency; ↓: decreased significantly; ↘: probably decreased; ↔ : no effect, or effect unclear; -: no data available, ↗: probably increased; ↑: increased significantly

### Macrophages

Macrophages arise from monocyte precursors in the bloodstream and differentiate into tissue-specific macrophages upon entering tissues. Their primary functions include phagocytosis and regulation of inflammatory responses. However, macrophages in aged organisms exhibit several functional impairments, which contribute to a weakened immune system and increased susceptibility to inflammation and infection. Notably, both aging and zinc deficiency are associated with an increased number of macrophages [217, 218] and monocytes [167, 172, 219, 220].

As organisms age, macrophages display reduced phagocytic activity, compromising innate immune defense mechanisms. This impairment is associated with increased survival rates of pathogens, such as *Streptococcus pneumoniae*, and a slight rise in pro-inflammatory cytokine levels, as demonstrated in a mouse model [221]. Moreover, diminished phagocytic function in macrophages is linked to delayed wound healing [222] and impaired clearance of apoptotic cells, which contributes to chronic inflammation in aged tissues [223].

Aging also affects macrophage polarization, the process by which macrophages differentiate into either pro-inflammatory M1 or anti-inflammatory M2 phenotypes (Shapouri-Moghaddam et al. 2018). In elderly organisms, the balance between these phenotypes may shift, leading to heightened inflammation. Patients with chronic liver disease often have zinc deficiency. In a mouse model of liver fibrosis, zinc supplementation significantly suppressed fibrosis and inflammation, reduced collagen deposition, and decreased macrophage infiltration, together with inhibiting hepatic stellate cells. Zinc selectively inhibited M1 macrophage polarization and the production of M1-related inflammatory cytokines, including iNOS, monocyte chemotactic protein-1 (MCP-1), and TNF-α. Zinc´s anti-inflammatory effects were also linked to inhibition of Notch1 pathway, which regulates M1 macrophage polarization [224]. Moreover, both zinc deficiency and zinc supplementation in THP-1-derived macrophages were observed to enhance M1 polarization, while concurrently suppressing M2 polarization. The inhibitory effect of zinc supplementation on M2 polarization suggests benefits for the treatment of M2-dominated conditions, such as allergic asthma, or, more commonly observed in the aging population, cancer [225].

Mitochondrial dysfunction is another key factor in the decline of macrophage function with aging. NAD^+^, a cofactor critical for many enzymatic processes in macrophages, declines with age. Insufficient NAD^+^ synthesis leads to reduced mitochondrial respiration and phagocytosis, contributing to heightened inflammatory responses. In elderly human monocyte-derived macrophages (MDMs), increased oxygen consumption and decreased activity of mitochondrial complexes I and II have been observed, along with reduced NAD^+^ synthesis, which may explain the overall decline in immune function with aging [226].

Furthermore, macrophages from aged individuals exhibit impaired mitophagy, the process of recycling damaged mitochondria. This impairment leads to the accumulation of dysfunctional mitochondria, exacerbating oxidative stress and inflammation [227]. The decreased expression and signaling of TLRs, essential for recognizing pathogen-associated molecular patterns (PAMPs), further impair the ability of aging macrophages to phagocyte apoptotic cells and respond to pathogens. Reduced TLR signaling also diminishes cytokine secretion, further weakening the immune response [228].

Cytoplasmic, bioavailable zinc is essential for IL-1β production in activated human monocytes and macrophages, acting downstream of glycolysis, which is induced by the mammalian target of rapamycin complex 1 (mTORC1). Zinc enhances the phosphorylation of S6 kinase (S6 K) by mTORC1 through inhibiting protein phosphatase 2 (PP2 A), a phosphatase that dephosphorylates S6 K. The activity of PP2 A is increased in zinc deficiency [229, 230]. In patients with rheumatoid arthritis (RA), a common disease in the aged population, decreased expression of the zinc transporter Zip8 in monocytes leads to lower intracellular zinc levels. Notably, there is a positive correlation between Zip8 expression and more severe RA clinical parameters [230].

Zinc transporters, particularly Zip proteins, also play a role in macrophage polarization. In a study on hepatocellular carcinoma (HCC), Zip2 and Zip9 were upregulated in M2-polarized macrophages, while Zip9 was slightly downregulated in M1-polarized macrophages. Tumor-associated macrophages derived from human liver cancer tissues exhibited decreased transcription of Zip9 compared to adjacent, non-cancerous tissues. Dysregulated Zip expression, particularly Zip9, may influence immune responses in HCC, contributing to cancer pathogenesis and progression [231].

Zinc plays a critical role in macrophage-mediated pathogen defense. In a study on the fungal pathogen *Histoplasma capsulatum*, macrophages treated with the glucose analog 2-deoxy-D-glucose (2-DG) showed reduced exogenous zinc import, resulting in zinc starvation, which led to fungal death. This reduction in zinc import was likely due to impaired zinc transporter activity, emphasizing the importance of zinc in controlling infection [232]. Additionally, zinc supplementation increased the population of peritoneal macrophages in *Trypanosoma cruzi* infection models [233].

In chronic obstructive pulmonary disease, a disease with increased prevalence in aged individuals, zinc deficiency hinders the clearance of apoptotic epithelial cells by alveolar macrophages, contributing to persistent inflammation [234]. Zinc deficiency due to alcohol abuse similarly impairs alveolar macrophage function, reducing phagocytic activity and compromising immune response [235]. In alcohol fed rats, zinc supplementation could improve phagocytic function [236]. Moreover, zinc transporters such as Zip7 have a more direct impact on macrophage activity. In Zip7-knockdown THP-1 cells, phagocytosis is notably less efficient, through this deficiency can be reversed by zinc supplementation. Furthermore, a deficiency in Zip7 shifts macrophage activation towards an M2 phenotype, as evidenced by increased expression of the M2 marker CD206 and reduced levels of the M1 marker NOS2, along with lower production of proinflammatory cytokines such as TNF-α and IL-6. Again, zinc supplementation effectively restores these defects, underlining the role of zinc, and specifically Zip7, in supporting phagocytosis and macrophage activation [14].

In addition to supporting their function, zinc is critical for preventing macrophage apoptosis. In a genetic mouse model, the loss of zinc transporter Zip10 leads to reduced zinc levels in macrophages, triggering p53-dependent apoptosis [74]. Comparatively, aged macrophages are more sensitive to apoptotic signals as well [237].

Zinc also plays a crucial role in the differentiation of myeloid cells, but its effects can vary depending on the context. For instance, low zinc levels can block the differentiation of HL-60 cells into macrophages when induced by phorbol 12-myristate 13-acetate (PMA), an effect that can be partially reversed by adding external zinc [238]. In contrast, zinc deficiency appears to enhance the differentiation and maturation of HL-60 cells into macrophages when stimulated by 1 α, 25-dihydroxyvitamin D D(3) (1,25D(3)) [218].

Zinc´s role in modulating oxidative stress and ROS production in immune cells is context dependent. In PBMCs, including monocytes, a reduction in zinc concentration, either through the use of the chelator TPEN or by removing zinc from the culture medium, leads to a significant increase in oxidative burst and phagocytosis following infection with gram-positive *S. aureus* [239]. Similarly, in a study on *E. coli-*infected rats, zinc supplementation increased in vivo superoxide production. However, in vitro, zinc inhibited superoxide production by macrophages derived from septic rats [240]. Additionally, zinc sequestration mechanisms can enhance ROS production in infected macrophages. For instance, in *Histoplasma capsulatum*-infected macrophages, GM-CSF activation induces the sequestration of labile zinc through MT expression. Zinc exporters such as ZnT4 and ZnT10 are upregulated, redirecting zinc away from phagosomes and into the Golgi apparatus. This zinc redistribution enhances H^+^ channel function within phagosomes and triggers ROS generation, which effectively halts *H. capsulatum* replication and improves fungal clearance [241].

Zinc also plays a crucial role in regulating inflammatory signaling pathways in monocytes and macrophages. The activation of TLR4 in monocytes and granulocytes induces a rapid rise in intracellular zinc levels. Chelating this zinc signal with the membrane-permeable chelator TPEN effectively blocks the activation of LPS-induced signaling pathways, including the p38 MAPK, extracellular-signal regulated kinases 1/2 (ERK1/2) and NF-κB pathways, ultimately inhibiting the release of proinflammatory cytokines such as TNF-α [18]. Moreover, LPS-induced stimulation of macrophages activates the JAK-STAT1 pathway through the release of IFN-β, resulting in the expression of iNOS. In this context, free intracellular zinc ions again serve as second messengers in LPS-dependent gene expression. Chelating zinc leads to an inhibition of both LPS- and IFN-β-mediated increases in STAT1 mRNA expression in RAW 264.7 macrophages [242]. Higher zinc concentrations, however, can suppress the release of other proinflammatory cytokines, such as IL-1β and TNF-α, by inhibiting phosphodiesterase activity, thus modulating the inflammatory response [243].

Zinc is essential for the production of IFNs, especially IFN-α, which plays a crucial role in antiviral immunity. Monocytes, alongside plasmacytoid dendritic cells, are major producers of IFN-α during viral infections, contributing to its antiviral and immunomodulatory properties [244]. The age-related decline in IFN-α production [245], which increases the risk of viral infections, highlights the significance of findings by Cakman et al., who demonstrated that in vitro supplementation of blood from elderly individuals with 15 µM zinc significantly enhanced IFN-α production [167].

**Fig. 4:**
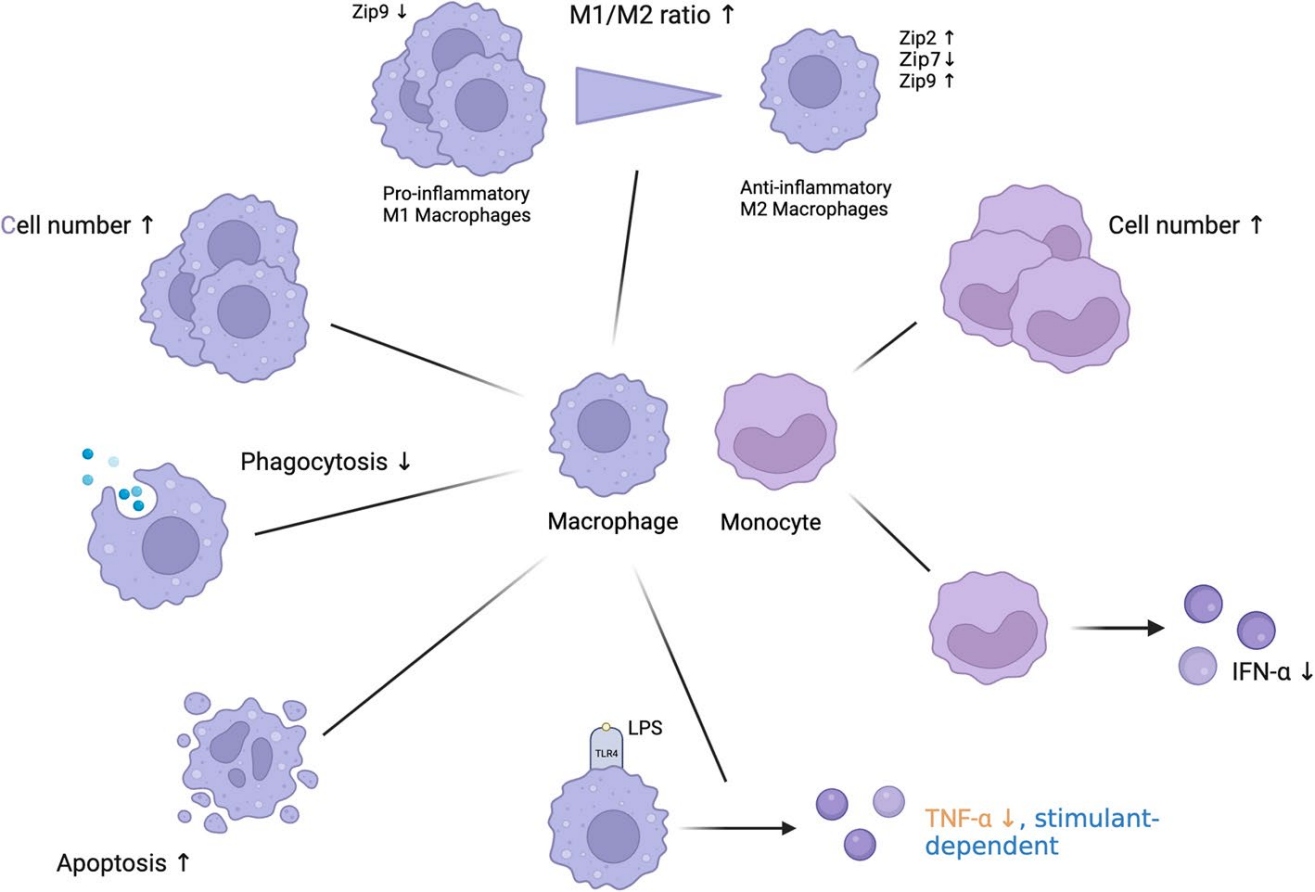
Macrophage and Monocyte Dysfunction in Aging and Zinc Deficiency.  : Alterations due to aging, but not described in zinc deficiency,  : Alterations due to zinc deficiency, but not described in aging, (Black) Alterations described in both, zinc deficiency and aging, (↑): increased, (↓): decreased Created in https://BioRender.com

In summary, zinc supports aging individuals by enhancing macrophage function, improving pathogen clearance, reducing chronic inflammation and aiding wound healing. It balances macrophage polarization, supports mitochondrial health and boosts immune signaling pathways (Fig. 4 and Table 7).

**Table 7:** Comparison of monocyte and macrophage counts and functions in aged individuals and zinc deficient state

Cell type	Characteristics	Alteration		References	
		**Elderly**	**ZD**	**Elderly**	**ZD**
**Monocytes**					
	Cell number	↗	↗	[167] ↗ [220] ↑	[172, 219] ↑ [246] ↓
	ROS production	↑	↔	[247] ↑	[239] ↑ [240] ↑↓
	IFN-α-production	↓	↓	[245] ↓	[167] ↓
**Macrophages**					
	Cell number (tissue resident)	↑	↘	[217] ↑	[248] ↗ [218] ↑
	Apoptosis	↑	↑	[237] ↑	[74] ↑
	Phagocytosis/Clearance	↓	↓	[221–223] ↓	[234, 236, 249] ↓
	M1/M2-ratio	↑	↑	[237] ↑	[224, 225, 246] ↓

ZD: Zinc deficiency; ↓: decreased significantly; ↘: probably decreased; ↔ : no effect, or effect unclear; -: no data available, ↗: probably increased; ↑: increased significantly

### Dendritic cells

Dendritic cells (DCs) play a pivotal role in the immune system by serving as professional antigen-presenting cells (APCs). They are essential for initiating and regulating immune responses. DCs capture antigens through surface receptors, process them into peptide fragments, and present these fragments as major histocompatibility complex (MHC)-peptide complexes on their surface. This process enables DCs to activate T cells, a critical step in the initiation of adaptive immune responses. Furthermore, DCs possess the ability to follow chemotactic signals, guiding them to immune-related tissues such as lymph nodes, where they interact with T cells, further underlining their crucial role in regulating immune responses [250].

DCs can be divided into two primary subtypes: plasmacytoid DCs (pDCs) and myeloid DCs (mDCs) [251]. Both subtypes have distinct roles in immune surveillance and response, with pDCs known for their ability to produce IFNs, while mDCs are more specialized in antigen presentation.

Aging has a profound impact on the function and population of DCs, particularly pDCs. In elderly individuals, there is a marked decline in the number of circulating pDCs. This decline is also associated with a decreased ability of these cells to migrate to lymph nodes, a crucial step for T cell activation [252, 253].

Elderly women exhibit a reduced number of circulating pDCs compared to younger women. Additionally, both pDCs and mDCs from older women show diminished responsiveness to TLR stimuli, including TLR7 and TLR8 activation [254]. Additionally, old DCs express lower levels of CCR7 and CCR9, resulting in decreased migratory activity, as demonstrated in studies on aged mice [255]. Despite these changes, the transendothelial migration of DCs remains unchanged compared to younger controls. Furthermore HLA-DR expression is reduced in the peripheral blood DCs of elderly subjects, which leads to diminished antigen presentation and contributes to the age-related decline in immune responsiveness [256].

Aging also affects the cytokine production of pDCs. Compared to healthy adults, pDCs from elderly individuals produce fewer type I IFNs in response to stimuli such as CpG and influenza virus [257]. Moreover, elderly pDCs are less capable of stimulating CD8^+^ T cells to produce perforin and granzyme, which are crucial for cytotoxic activity, and they are less efficient in promoting IFN-γ secretion by CD4^+^ and CD8^+^ T cells [258]. These functional declines are compounded by dysfunctional signaling in TLR2 and TLR4 pathways, further impairing immune responses in the elderly [259].

Aligned with the concept of inflammaging, DCs develop a more pro-inflammatory phenotype as they age [260–262]. In this context, zinc plays a crucial role promoting the tolerogenic phenotype in DCs and suppressing pro-inflammatory responses, thereby helping to restore a balance that is critical in aging immune systems [263, 264]. Zinc peroxide nanoparticles significantly induce a transition from immunogenic DCs to tolerogenic DCs in a mouse model of rheumatoid arthritis, thereby reducing T-cell response which drives immune progression of rheumatoid arthritis. The zinc nanoparticles reduced swelling of the ankle, improved mobility and pain reduction. However, there wase no prevention of bone destruction, but a reduced synovial inflammation [265]. Thereby zinc supplementation offers interesting potential to counteract age-related declines in DC function.

When exposed to zinc in *vitro*, DCs derived from bone marrow exhibited a tolerogenic profile, characterized by reduced surface MHC-II expression and increased levels of tolerogenic markers such as Programmed Death-Ligand (PD-L) 1, PD-L2 and the tryptophan degrading enzyme, Indoleamine 2,3-dioxygenase (IDO). Zn also suppressed the proinflammatory response to TLR ligands. Zn supplementation also shifted the Treg-Th17 balance towards Foxp3^+^ regulatory T cells (Tregs) [263, 264]. This shift can be attributed to Zn´s ability to inhibit STAT3 activation, which is crucial for Th17 differentiation. All this was shown in vivo with *Histoplasma capsulatum* fungal infections as well [263]. In aging populations, this balance can mitigate excessive inflammatory responses while enhancing regulatory pathways, reducing the risk of chronic inflammatory diseases.

Zinc supplementation also exerts beneficial effects in vivo. In zinc-deficient rats, zinc supplementation increased the number of DCs and mucosal cells producing secretory IgA while reducing the production of proinflammatory cytokines IL-6 and IFN-γ. This increase in DCs was observed even in rats with normal serum zinc levels [266]. Zinc deficiency also leads to a decrease in epidermal transforming growth factor (TGF)-β1, which in turn reduces the number of epidermal Langerhans cells, another important subset of DCs [267, 268]. In elderly individuals, maintaining zinc homeostasis could help preserve DC numbers and their critical roles in mucosal immunity, a key aspect of protecting against infections.

Zinc also affects DC survival and apoptosis. In mice, low concentrations of zinc stimulate ceramide formation, leading to DC apoptosis through a mechanism involving acid sphingomyelinase. However, LPS treatment was found to protect DCs from apoptosis, demonstrating the complex interplay between zinc and immune signaling [269]. Furthermore, LPS stimulation suppresses the expression of zinc importers (Zip6 and Zip10) while upregulating zinc exporters (ZnT1, ZnT4 and ZnT6), resulting in a net reduction of intracellular zinc. This decrease in intracellular zinc is associated with increased surface expression of MHC-II and CD86, key markers of DC maturation. This effect of LPS-induced zinc depletion can be replicated using zinc-chelating agents, and zinc supplementation inhibits the movement of MHC-II-positive vesicles, empathizing the importance of zinc homeostasis in DC function [270]. For aging individuals, supplementation may prevent these zinc-dependent disruptions, thereby sustaining optimal DC maturation and antigen presentation capacities.

DCs are also capable of synthesizing 1,25-dihydroxy vitamin D (1,25(OH)2D), which plays an essential role in the induction of Tregs. Animal studies suggest that high concentrations of 1,25(OH)2D are necessary to generate Tregs when supplemented, but this can lead to adverse side effects such as hypercalcemia. However, overexpression of 1α-hydroxylase in DCs enables production of 1,25(OH)2D in Treg-inducing concentrations without causing hypercalcemia. Interestingly, the addition of zinc in non-toxic concentrations significantly enhances the Treg-inducing capacity of DCs, offering a promising therapeutic strategy for immune-mediated diseases [271].

In summary, zinc deficiency worsens age-related declines in DC function, leading to reduced antigen presentation, impaired cytokine production, and weakened immune response. Effects that can possibly be improved by zinc supplementation (Table 8).

**Table 8:** Comparison of dendritic cell counts and functions in aged individuals and zinc deficient state

Cell type	Characteristics	Alteration		References	
		**Elderly**	**ZD**	**Elderly**	**ZD**
**Dendritic cells**					
	Immunogenic/tolerogenic ratio	↑	↑	[260–262] ↑	[263, 264] ↑
	Antigen presentation	↔	↑	[258, 272] ↔	[270, 273] ↑
Plasmacytoid dendritic cells					
	Cell number	↘	-	[274–276] ↓ [220] ↔	-
	IFN production	↓	↓	[257, 258] ↓	[266] ↓
	T cell stimulation	↓	↑	[258, 259] ↓	[270] ↑
Myeloid dendritic cells					
	Cell number	↘	↓	[274, 276] ↓ [220, 275] ↔	[277] ↓
**Langerhans cells**					
	Cell number	↓	↓	[278–280] ↓	[268, 281, 282] ↓

ZD: Zinc deficiency ↓: decreased significantly; ↘: probably decreased; ↔ : no effect, or effect unclear;

-: no data available, ↗: probably increased; ↑: increased significantly

## Changes in the adaptive immune system

### T cells

Immunosenescence leads to significant changes in T cell populations and their effectiveness. This process involves a decrease in naïve T cells and an accumulation of memory T cells, impairing the immune system´s ability to respond to novel or fast-mutating pathogens, such as influenza virus while preserving responsiveness to previously encountered ones [283]. More specifically, in the periphery of aged people there is a rise in CD45R0^+^ memory T cells and a reduction in CD45RA^+^ naïve T cells [284].

One of the primary reasons for the decline in naïve T cells is thymic involution, a process where the thymus, responsible for producing new T cells, shrinks over time, thereby compromising the adaptive immune response [285–287].

Thymic epithelial cells also secrete thymulin, a hormone essential for T cell development and regulation, which depends on zinc to fold into its active form. Zinc acts as a cofactor, stabilizing thymulin's structure and ensuring its proper function in immune regulation. Without zinc, thymulin remains biologically inactive, impairing its role in T-cell differentiation and immune modulation [288]. As thymulin production decreases with age, the overall effectiveness of T cells in immune responses is further reduced [289, 290]. Thymic atrophy in the elderly is often associated with zinc deficiency, contributing to reduced activity of thymulin, lower production of naïve T cells and increased apoptosis in thymocytes [291, 292]. One reason for the increased probability of apoptosis in zinc deficient thymocytes is the higher expression of the protein p56^lck^ [293, 294]. In a mouse model, zinc supplementation has been shown to reverse certain age-related thymic defects and enhance thymopoiesis [295, 296]. Additionally, mild zinc deficiency in humans and mice has been shown to reduce serum thymulin activity, which can be restored through zinc supplementation both in vivo and in vitro [297]. In aged mice, zinc supplementation has been observed to enhance thymulin secretion, potentially contributing to improved thymic function [290, 298].

Aging is also associated with an increase in mitochondrial content in memory CD4^+^ T cells, which leads to the generation of ROS and subsequently promotes the expression of proinflammatory cytokines [299]. This rise in proinflammatory cytokines is particularly evident in memory T cells, which display a senescence- associated phenotype [300–302].

Zinc supplementation in zinc-deficient elderly individuals, such as those in nursing homes, has been shown to improve immune function by enhancing anti-CD3/CD28 and PHA-stimulated T cell proliferation, as well as increasing the number of peripheral T cells [303].

Zinc is also critical for the activation of T cells. Following T cell receptor (TCR) activation, zinc ions enter the cell via zinc transporters such as Zip6 and Zip8, facilitating the signaling pathways necessary for T cell activation and proliferation [69, 304]. This influx of zinc enhances the production of proteins required for TCR signaling, such as lymphocyte protein tyrosine kinase (LCK) and PKC, which are essential for downstream immune responses [305, 306].

Zinc deficiency can severely impair immune responses. It leads to reduced T cell proliferation and diminished cytokine production such as IL-2 and IFN-γ [307, 308], a condition seen in aged individuals as well [309, 310], resulting in an increased susceptibility to infections.

There are also specific alterations in T cell subsets associated with aging. One notable change is the accumulation of CD8^+^ T cells that express senescence markers such as CD57 and the T-cell immunoreceptor with Ig and ITIM domains (TIGIT) [311]. Additionally, aged CD70^+^ T cells often exhibit an over-activated phenotype, characterized

 by elevated levels of inhibitory receptors, including programmed cell death protein (PD-1), 2B4, and lymphocyte-activation gene 3 (LAG-3) [312]. These over-activated T cells demonstrate increased susceptibility to apoptosis and produce higher levels of cytokines. This heightened susceptibility to apoptosis is not unique to aged CD70^+^ T cells. T cells in general are more prone to apoptosis [313, 314]. Furthermore, zinc deficiency exacerbates apoptosis in T cell progenitors by disrupting the Bcl-2/Bax pathway, leading to elevated rates of cell death. Conversely, zinc supplementation can mitigate this effect by inhibiting caspase-3, −6, −7 and −8, thereby reducing apoptosis [315].

**Fig. 5:**
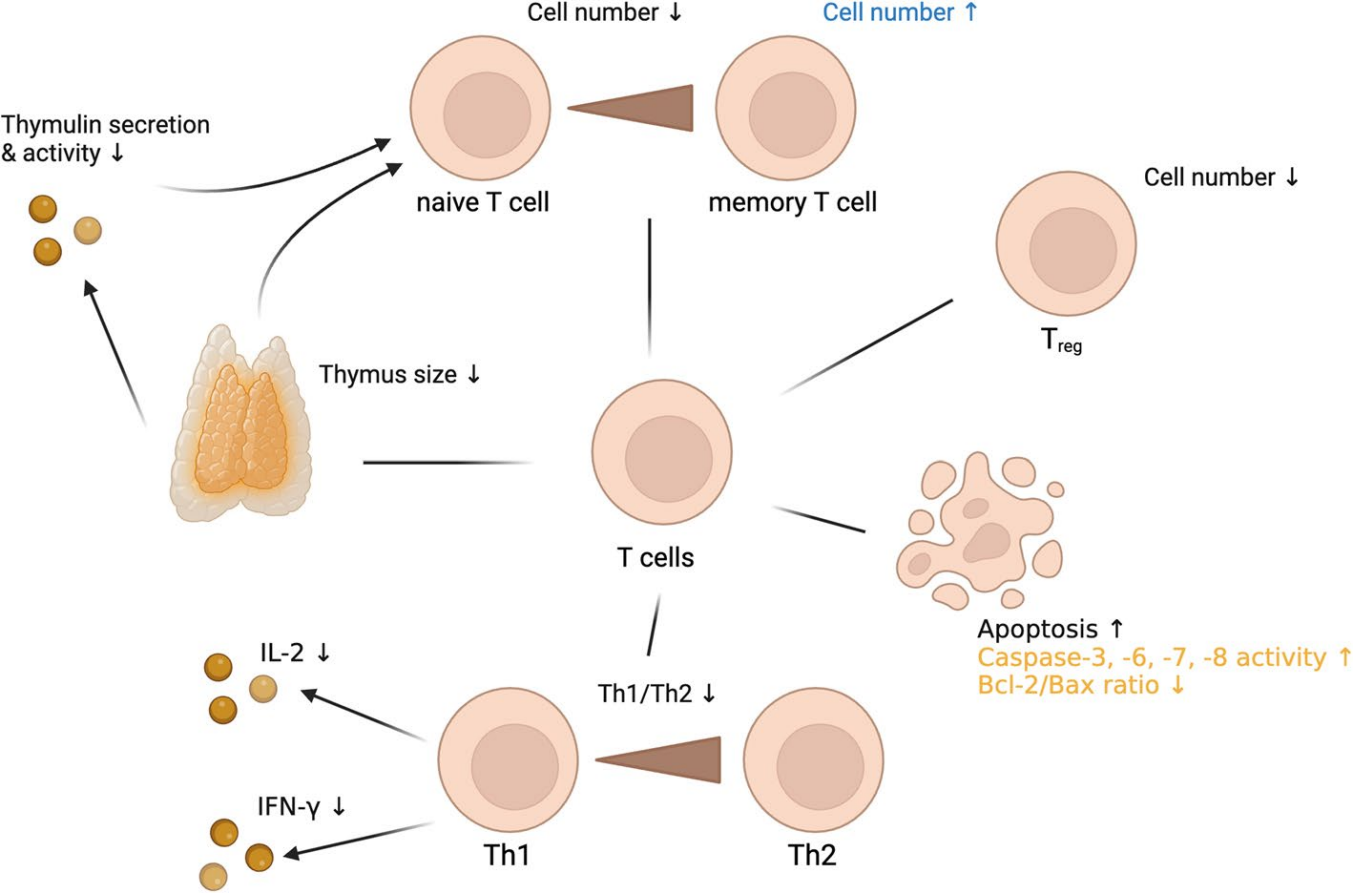
T Cell Dysregulation in Aging and Zinc Deficiency.  : Alterations due to aging, but not described in zinc deficiency,  : Alterations due to zinc deficiency, but not described in aging, (Black) Alterations described in both, zinc deficiency and aging, (↑): increased, (↓): decreased Created in https://BioRender.com

In addition to its impact on T cell function, zinc plays a crucial role in regulating the balance between T helper cell subsets, particularly the Th1/Th2 ratio. As individuals age, the immune response shifts towards a Th2-dominant profile, reducing the body´s ability to combat intracellular pathogens [15, 316]. Zinc supplementation can restore this balance by enhancing Th1 responses, by increasing the production of Th1 cytokines such as IFN-γ and IL-2 and promoting the expression of the transcription factor T-bet [229, 317]. Conversely, zinc deficiency shifts the immune response further towards a Th2-dominant profile, going along with the reduction of Th1 cell cytokines, such as IFN-γ, IL-2 and TNF-α. Interestingly Th2 cytokines like IL-4, IL-6 and IL-10 remained unchanged [318]. In cases of zinc deficiency, even short-term supplementation of in average 6 days can significantly improve immune function by decreasing CREMα expression and thereby increasing IL-2 production [309]. Zinc supplementation in elderly individuals has been shown to reduce the incidence of infections by increasing the numbers of CD4^+^ T cells and cytotoxic T lymphocytes through enhanced synthesis of IL-2 and soluble IL-2 receptor (sIL-2R) [319], (Fig. 5 and Table 9).

**Table 9:** Comparison of T cell counts and functions in aged Individuals and zinc-deficient state

Cell type	Characteristics	Alteration		References	
		**Elderly**	**ZD**	**Elderly**	**ZD**
**T cells**					
	Thymus size	↓	↓	[285–287] ↓	[334, 335] ↓
	Secretion/activity of thymulin by thymic epithelial cells	↓	↓	[289, 290] ↓	[290, 292, 297, 298] ↓
	Apoptosis	↑	↑	[313, 314] ↑	[315] ↑
	Naïve T cells: Cell number	↓	↘	[308, 336–339] ↓	[308] ↓ [315, 340] ↘
	Memory T cells: Cell number	↑	↑	[338, 341, 342] ↑	[343] ↗ [344] ↑
	Tregs: Cell number	↓	↘	[322] ↓	[323] ↗ [324] ↓
	Th1/Th2 ratio	↓	↓	[15, 316] ↓	[15, 318, 343] ↓
	IL-2 and IFN-γ production	↓	↓	[309, 310] ↓	[307–309, 318] ↓

ZD: Zinc deficiency; ↓: decreased significantly; ↘: probably decreased; ↔ : no effect, or effect unclear; -: no data available, ↗: probably increased; ↑: increased significantly

Proton-pump inhibitors, such as pantoprazole, are commonly used to treat various gastrointestinal diseases in elderly patients. However, pantoprazole has been shown to reduce the expression of zinc transporter Zip8 and increase the expression of the transcription factor CREMα. This alteration results in downregulation of IFN-γ and IL-2 expression, leading to an increased risk of infections [320].

The role of zinc in modulating Tregs is another key factor in its immunoregulatory effects. Tregs are responsible for maintaining immune homeostasis by suppressing overactive immune responses, preventing autoimmune reactions, and ensuring tolerance to self-antigens [321]. The levels of Tregs in the circulating blood are diminished in older individuals [322]. During zinc deficiency, there is an increase in Treg cell numbers. However, these cells are likely nonfunctional as they express low levels of microRNS-146, which is critical for their proper function upon stimulation [323].

Zinc promotes Treg differentiation by inhibiting the activity of Sirtuin 1 (Sirt1), an enzyme that deacetylates Foxp3, a modification that leads to its ubiquitination and subsequent degradation. Foxp3 is a key transcription factor for Treg development [324]. Moreover, treatment of mixed lymphocyte cultures (MLC) with zinc and TGF-β1 triggers intracellular zinc signaling, which, in combination with increased Smad2/3 activation, enhances Foxp3 expression. This results in a mitigated allogenic immune response in MLC, suggesting that zinc can elevate Treg cells in adverse immune reactions [325]. Furthermore, an increased number of Tregs in PBMCs from atopic individuals is associated with the suppression of allergic hyperresponsiveness (Th2-mediated) and allogenic reactivity (Th1-mediated) [326].

Vitamin D3 and zinc, when administered together, more effectively induce regulatory T cells and suppress IFN-γ production in mixed lymphocyte cultures compared to treatment with either Vitamin D3 alone. Vitamin D3 enhances intracellular zinc levels by upregulating the expression of Zip13. Consequently, lower concentrations of Vitamin D3 and zinc may serve as effective treatment options, thereby minimizing the side effect associated with higher doses of Vitamin D3 [327].

The role of zinc is not universally beneficial. In the context of cancer, high dietary zinc intake has been shown to promote tumor progression by fostering a pro-tumorigenic environment mediated by T cells. Conversely, zinc deficiency or chelation of tissue zinc improves anti-tumor immunity and enhances responses to immunotherapies such as αPD-1, particularly in melanoma models [328].

Moreover, zinc supplementation has been shown to reduce the vulnerability of tumor cells to TNF-dependent lysis mediated by CD8^+^ T cells, whereas zinc chelation enhances this susceptibility. Additionally, the loss of the transcription factor CBFβ in tumor cells disrupts zinc homeostasis pathways, further impairing their responsiveness to TNF-dependent cytotoxicity [329].

Increased Th17 cell numbers have been observed in the elderly [322]. In both mouse models of autoimmune encephalomyelitis and human subjects, supplementation with zinc aspartate has been found to suppress the development of Th17 cells through the degradation of STAT-3, which may help alleviate the impact of Th17-driven autoimmune disorders [330, 331]. Zinc also inhibits the differentiation of Th17 cells by interfering with the IL-6/STAT3 signaling pathway and preventing the phosphorylation of IL-1 receptor-associated kinase 4 [332]. Moreover, zinc suppresses Th17 cells by inhibiting glutaminolysis, which serves as the primary energy source for these cells, thereby lowering the risk of autoimmune diseases, such as autoimmune encephalomyelitis [333]. 

### B cells

The overall number of B cells remains relatively stable throughout most of human lifespan, with a decline only observed in advanced old age, long after the initial signs of immune system inefficacy become apparent [345, 346]. In contrast, murine studies show that while the total number of peripheral B cells in mice remains consistent as they age, the influx of new naïve B cells into peripheral tissues decreases in older mice. This decline is attributed to the slower turnover rate of splenic B cells in aged mice, allowing them to survive longer and signaling to newly generated B cells that peripheral niches are fully occupied [347, 348]. Some researchers also suggest that there is a reduced ability of naïve B cells from older mice to populate peripheral compartments [349]. The reduced influx of new B cells is also due to bone marrow cells having a reduced capacity for B cell generation in culture compared to bone marrow of younger mice [350]. Although aging does not inherently alter individual hematopoietic stem cells (HSCs), it does shift the clonal composition of the HSC pool. In aged mice, there is a decrease in lymphoid-biased HSCs and an accumulation of long lived myeloid-biased HSCs, reducing B cell production capacity [351, 352]. Additionally, the absolute number of B cell precursors in the bone marrow declines with age, particularly during adolescence [353], though B lymphopoiesis continues throughout adulthood [354]. Also, in vitro studies indicate that the responsiveness of pro-B cells to IL-7 is compromised in aged individuals [355], and IL-7 release from stromal cells in the bone marrow also declines with age [356], leading to reduced pro B-cell proliferation.

In elderly humans, there is a significant decline in CD27^dull^ memory B cells, a subset crucial for bridging innate and adaptive immunity. Instead, highly differentiated, antigen-selected CD27^bright^ memory cells dominate. After in vitro stimulation with CpG, B cells from older individuals produce significantly lower levels of IgM and IgA antibodies compared to younger individuals, highlighting a diminished capacity to respond to novel pathogens while maintaining responsiveness to frequently encountered antigens [357]. This is empathized by the decrease of naïve B cells and increase of memory B cells in elderlies [358]. Furthermore, there is an increase in memory B cells lacking the activation marker CD27 in peripheral blood of elderly individuals, a phenomenon also observed in chronic viral infections and autoimmune diseases such as systemic lupus erythematosus [359, 360].

There is also a decline in the IgM memory compartment with age [361], which may contribute to a weakened immune response to polysaccharide antigens, increasing susceptibility to bacterial infections like pneumococcal disease [362–364]. This is particularly relevant since the reduction of IgM levels in serum has been linked to decreased opsonophagocytic function [365].

In human studies examining the V_H_ chain repertoire following pneumococcal vaccination, older adults exhibit reduced oligoclonality and a lower frequency of somatic mutations compared to younger individuals [366]. A decline in activation-induced cytidine deaminase (AID), necessary for class switch recombination (CSR) and somatic hypermutation, is observed in elderly individuals. This decrease is attributed to lower stability of E47, a transcription factor for AID, and E47 mRNA. Higher levels of microRNAs, specifically miR-155 and miR-16, in aged B cells may inhibit AID and E47 expression, reducing protein synthesis [367]. Additionally, in vitro studies have demonstrated that zinc chelation inhibits AID activity, suggesting that age-associated changes in zinc homeostasis might further exacerbate the reduction in AID functionality [368]. However, Banerjee et al. [369] demonstrated that somatic hypermutation rates are similar in both young and elderly individuals, suggesting that the increased number of mutations in Ig genes seen in older adults [370] likely results from cumulative exposure over time rather than an inherently altered mutation process.

Research on repeated influenza vaccinations in elderly individuals suggests that memory B cell generation remains intact, though serum antibody titers increase less after vaccination compared to younger controls. Despite this impairment in antibody production, protective titers were still achieved.

 [371]. A reason for the reduced production of antibodies could be the notable decline in the presence of long-lived antibody-secreting plasma cells that recirculate within the bone marrow in older adults [372]. Furthermore, antibodies generated in elderly individuals are often less protective than those produced by younger ones. This is demonstrated by a decreased ability to opsonize bacteria-derived polysaccharides in vitro after vaccination [373].

Finally, aging induces a shift in baseline serum antibody specificity, with an increased prevalence of autoreactive antibodies, such as those targeting cardiolipin, DNA, and antinuclear antibodies (ANA), as well as rheumatoid factor. While this rise in autoreactivity is associated with autoimmune diseases like vasculitis, it does not necessarily result in immune pathology [374].

Zinc deficiency reduces the population of naïve B cells, which are essential for adaptive immunity [219]. This reduction is largely due to the disruption of lymphopoiesis, as zinc deficiency leads to a significant decline in pre-B cells [291]. Further research has shown that in zinc-deficient mice, while T-cell lymphopoiesis is significantly reduced, B cell development and maturation in the bone marrow are less affected [375]. However, the reduction in pre-B cells during zinc deficiency mirrors the effect observed in T cells and is associated with altered glucocorticoid metabolism and increased apoptosis, highlighting shared mechanisms of immune impairment under zinc deficiency [376].

The B cell receptor (BCR), essential for both immature and mature B cell activation and proliferation, is another area where zinc plays a pivotal role. The zinc transporter Zip10 is critical for modulating BCR signal strength [377] and preventing apoptosis in B cells, indicating zinc´s influence on humoral immunity through its regulation of BCR signaling [378].

Zinc deficiency also affects intracellular signaling pathways necessary for B cell function. Specifically, it hinders the activation of protein PKC in response to mitogens, as zinc deficiency prevents PKC´s translocation to the cell membrane. This impairment leads to a reduced proliferative response in B lymphocytes [379]. Moreover, zinc deficiency alters cytokine-mediated signaling, enhancing B cell proliferation in response to IL-6 and IL-2 but reducing it following IL-4 stimulation. This results in decreased STAT6 phosphorylation in IL-4 pathways and increased STAT3 phosphorylation in IL-6 pathways, emphasizing the role of zinc in maintaining balanced immune responses [380].

**Fig. 6:**
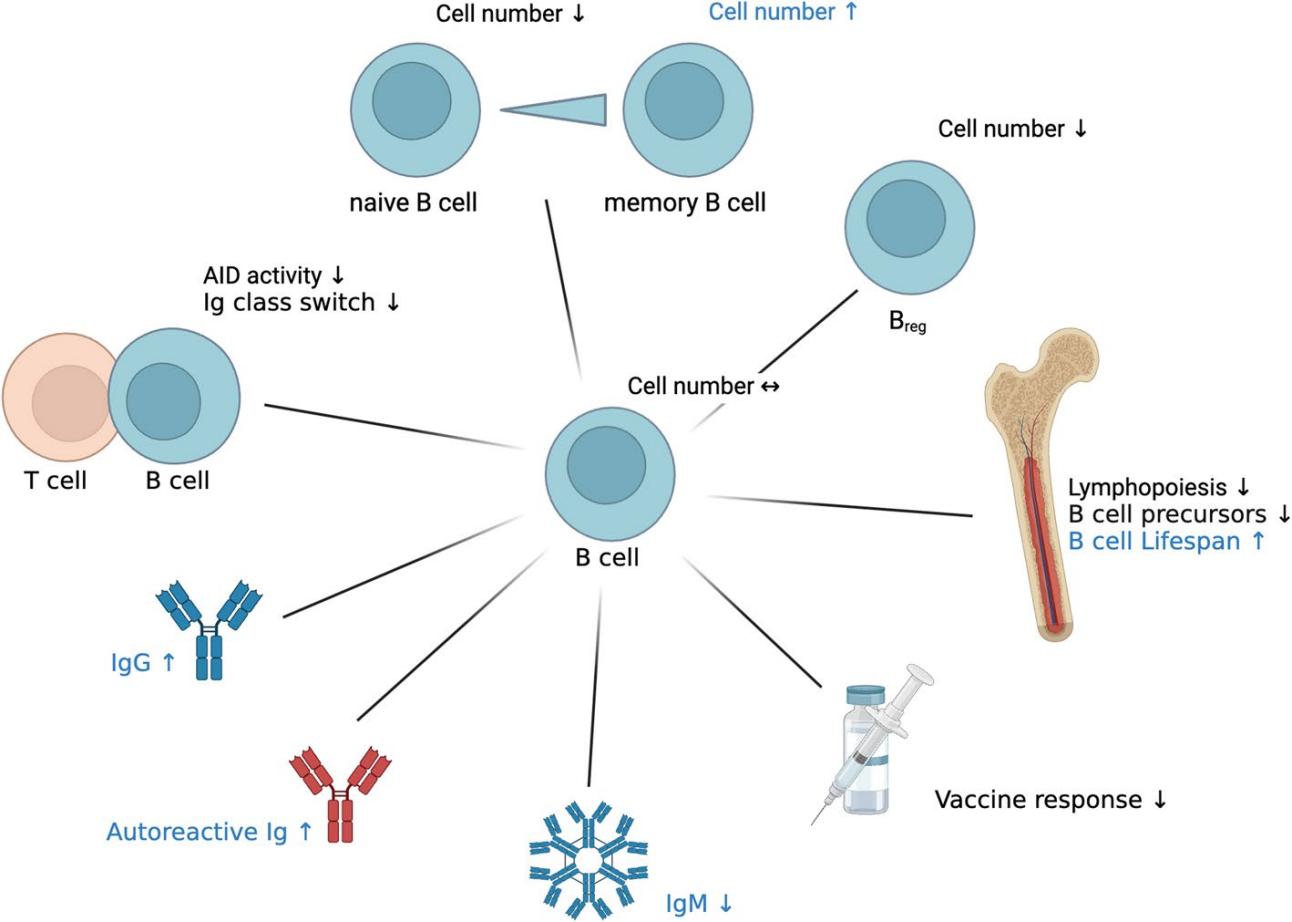
B Cell Alterations in Aging and Zinc Deficiency.  : Alterations due to aging, (Black) Alternations due to both, zinc deficiency and aging, (↑): increased, (↓): decreased Created in https://BioRender.com

Furthermore, regulatory B cells (Bregs), identified as CD19^+^IL-10^+^ B cells, play crucial role in modulating immune responses by secreting the anti-inflammatory cytokine IL-10. By fostering an anti-inflammatory environment, Bregs help mitigate excessive pro-inflammatory responses, offering potential benefits for managing conditions such as allergies, asthma, and autoimmune diseases. This makes supporting Breg function especially important in elderly individuals, as it could help counteract the inflammatory imbalances characteristic of aging. Importantly, zinc levels significantly influence Breg production, with zinc deficiency leading to reduced Breg generation from purified B cells [381], (Fig. 6 and Table 10).

**Table 10:** Comparison of B cell counts and functions in aged individuals and zinc deficient state

Cell type	Characteristics	Alteration		References	
		**Elderly**	**ZD**	**Elderly**	**ZD**
**B cells**					
	Lymphopoiesis	↓	↓	[347, 350] ↓	[219] ↓
	Naïve B cells: Cell number	↘	↓	[391] ↔ [358] ↓	[219] ↓ [392] ↘
	Memory B cells: Cell number	↗	-	[358] ↗ [393] ↔	-
	Bregs: cell number	↘	↓	[394, 395] ↘	[381] ↓
	Immunoglobulin class switch/AID	↓	↓	[396] ↓	[368, 397] ↓
	Serum IgG	↑	-	[398–400] ↑	-
	Serum IgM	↘	-	[357, 400] ↘	-
	Autoreactive antibodies	↑	-	[374] ↑	-
	Vaccination response	↓	↓	[371, 383, 401] ↓	[16, 382, 383, 388] ↓

*ZD* Zinc deficiency, ↓: decreased significantly; ↘: probably decreased; ↔ : no effect, or effect unclear, -: no data available, ↗: probably increased, ↑: increased significantly

In the context of vaccination, zinc supplementation has shown promising effects, particularly in elderly populations. For instance, zinc treatment improved the IgG antibody reaction to the tetanus vaccine in older adults [16]. Furthermore, in a study on pneumococcal vaccination, elderly individuals with a stronger immune response had significantly higher serum zinc levels [382]. Conversely, elderly chronic hemodialysis patients who exhibited an inadequate immune response to active diphtheria vaccination were found to have significantly lower serum zinc levels compared to those who responded effectively [383]. Zinc´s importance extends to prenatal health as well. Zinc deficiency during pregnancy has been found to impair offspring´s antibody response and T cell proliferation following hepatitis B vaccination. Additionally, maternal zinc deficiency suppresses the secretion of IFN-γ from splenocytes and reduces IFN-γ expression in both CD4^+^ and CD8^+^ T cells [384]. The timing of zinc supplementation is also critical for optimal vaccination outcomes. Research has shown that administering high doses of zinc concurrently with vaccination may result in suboptimal effects, whereas pre-vaccination zinc supplementation offers more effective enhancement of the antibody response [16, 385–387].

More recent studies have identified a correlation between free zinc levels and the strength of antibody responses, including the potency of neutralizing antibodies [388]. This connection is particularly significant given that antibody titers after vaccination decline more rapidly in the elderly [389], highlighting the potential role of zinc in supporting more robust and sustained immune responses in aging populations.

In studies of human B cells immortalized by Epstein-Barr virus, alterations in zinc transporter expression were observed. Specifically, there was an increase in the endoplasmic-reticulum-to-cytoplasm Zip7 mRNA and protein expression as well as phosphorylation, leading to elevated intracellular free zinc levels. These changes, however, were not limited to immortalized cells. Similar elevations were noted in vitro in activated B cells marked with CD69, suggesting that both activation and proliferation contribute to increased intracellular zinc [390]. Moreover, mutations in the Zip7 gene have been linked to a novel autosomal recessive disease characterized by agammaglobulinemia and early-onset infections, highlighting the crucial role of Zip7 in B cell development and immune function, as confirmed through CRISPR-Cas9 mutagenesis in mice [44].

## Age related diseases

As highlighted earlier, immunosenescence and inflammaging contribute significantly to an increased risk of infections, cancer, and autoimmune diseases in aging individuals. For example, people over the age of 65 represented more than 68% of new cancer cases in 2019 [402], and the incidence of *Clostridium difficile* infections was more than threefold higher among elderly individuals compared to younger adults [403].

Additionally, the interplay between COVID-19, aging, and zinc homeostasis has garnered considerable attention, given the heightened vulnerability of older adults to severe COVID-19 outcomes. In the U.S., over 75% of deaths due to COVID-19 occurred among individuals aged 65 and older, with more than 53% of these deaths occurring in those over 75 years of age [404]. Zinc plays a pivotal role in immune function, and its deficiency has been linked to worse outcomes in COVID-19 patients. Lower serum zinc levels have been shown to correlate with more severe symptoms, such as acute respiratory distress syndrome (ARDS) and prolonged hospital stays [405, 406]. Furthermore, patients who did not receive zinc supplementation experienced longer recovery times, worse clinical presentations, and higher mortality rates compared to those who received supplemental zinc [407]. These findings underscore the critical role of zinc in modulating immune responses and mitigating the severity of COVID-19 in aging populations.

While the relationship between zinc and COVID-19 has been extensively reviewed in the literature [408], this discussion will shift focus to other age-related diseases and the broader consequences of zinc deficiency, which is not only linked to immune dysfunction but also plays a significant role in other age-associated conditions, where immunological processes are not the sole contributors. These include a range of chronic, degenerative and metabolic diseases, highlighting the indispensable role of zinc in supporting overall health and resilience during the aging process.

### Age-related macular degeneration

Age-related macular degeneration (AMD) is the primary cause of blindness among older adults in industrialized countries. In 2015, AMD affected approximately 67 million individuals in Europe, with projections indicating an increase to 77 million by 2050 [409]. This acquired, degenerative condition is characterized by drusen deposits that interfere with the retinal pigment epithelium (RPE) and Bruch´s membrane, specifically affecting the macula, which is essential for central, detailed, and color vision [410]. AMD progresses in two main forms: the dry (non-vascular or early-stage) type, marked by drusen beneath the RPE, and the wet (neovascular or late-stage) type, characterized by choroidal neovascularization [411, 412]. The prevalence of AMD increases markedly with age, demonstrating a pronounced disparity between older and younger populations. Among individuals aged 64 years or younger, AMD is less common, with 9.3% exhibiting moderate signs. In contrast, this prevalence rises to 26.9% in those over 75 years, emphasizing the profound influence of aging on the progression of the condition [409].

While most AMD cases (90%) begin as the less aggressive dry type, around 10% progress to the vision-threatening wet form. However, no approved clinical therapy currently exists for dry AMD. Lifestyle changes such as avoiding smoking, following a healthy diet and taking antioxidant supplements are recommended. In contrast, wet AMD can be treated with anti- vascular endothelial growth factor (VEGF) antibodies to reduce neovascularization [413, 414]. Given that aging is the leading risk factor, alongside family history and specific genetic predispositions, understanding AMD´s development and potential interventions is vital for protecting vision in aging populations [415].

Several genes related to the complement system, including the gene of complement factor H (CFH) [416], age-related maculopathy susceptibility 2 (ARMS2) [417], and others, have been identified as risk factor for AMD. SNPs in the CFH gene can elevate the risk of developing AMD by up to six times [418]. Expanding on this, complement components C3 and C5 play a pivotal role in driving VEGF expression, a key factor in the angiogenesis characteristic of AMD [419]. Additional risk factors, such as, obesity, smoking, UV light exposure, and certain physiological traits (e.g., high blood pressure, light-colored irises, female sex), exacerbate AMD through oxidative stress mechanisms [420]. The retina possesses the highest metabolic rate of any human tissue, with the production of ROS being an inherent aspect of its normal function. Research indicates that RPE cells from individuals with AMD exhibit elevated ROS levels and an increased susceptibility to oxidative stress compared to RPE cells from non-AMD donors [421]. Zinc plays a critical protective role in maintaining retinal health, particularly in the RPE. It mitigates H_2_O_2_-induced damage, enhances SOD activity [422], and activates the antioxidant response element (ARE)-Nrf2 pathway [423], thereby protecting RPE cells against elevated ROS levels.

Zinc levels in the neural retinal decline significantly with advanced age, whereas concentrations remain stable in the RPE [424] This decline is particularly relevant given that zinc is stored in key retinal cells, including ganglion, horizontal, amacrine, and Müller cells [425, 426]. In AMD, RPE/choroid zinc levels are reduced by around 24% in humans, while zinc deposits are paradoxically high in drusen, possibly affecting CFH oligomerization [427, 428].

Due to zinc depletion in AMD-affected RPE, dietary zinc supplementation is a promising intervention to prevent disease progression. Large-scale trials, notably AREDS and AREDS2, support zinc´s efficacy in slowing AMD progression, especially in combination with antioxidants such as vitamins C and E and β-carotene [429–431]. Studies such as the Rotterdam Eye Study and the Blue Mountains Eye Study also observed that increased dietary zinc intake was associated with a reduced risk of AMD [20, 432, 433]. However, zinc´s effectiveness appears to vary depending on genetic background, benefiting CFH allele carriers in the Rotterdam Study [434] but also more significantly benefiting ARMS2 carriers in AREDS participants [435, 436].

Beyond AMD risk reduction, zinc´s role in the complement system is of particular interest, as zinc promotes CFH oligomerization and inactivation, potentially modulating inflammation in AMD [428]. As part of the innate immune response, complement system dysregulation is implicated in AMD pathogenesis, with zinc able to bind CFH and other complement proteins to modulate inflammatory responses [437, 438]. Another important mediator, Zip7, facilitates zinc transfer from the endoplasmic reticulum to the cytoplasm within RPE cells, a process responsive to inflammatory cytokines such as IL-1β and TNF-α. By regulating barrier dysfunction in the RPE, Zip 7 may influence AMD pathogenesis, as shown by the protective effects of inhibiting Zip7-mediated zinc movement against these cytokines [439].

Additionally, AMD´s pathogenesis involves the loss of RPE-derived VEGF signaling and defenestration of usually high permeable choriocapillaries in atrophied RPE areas, which disrupts retinal permeability [440, 441]. Zinc supplementation has shown potential in restoring these retinal fenestrations by promoting the redistribution of plasmalemmal vesicle associated protein-1 (PV-1), facilitating nutrient and waste exchange and improving retinal health in AMD [442].

**Fig. 7:**
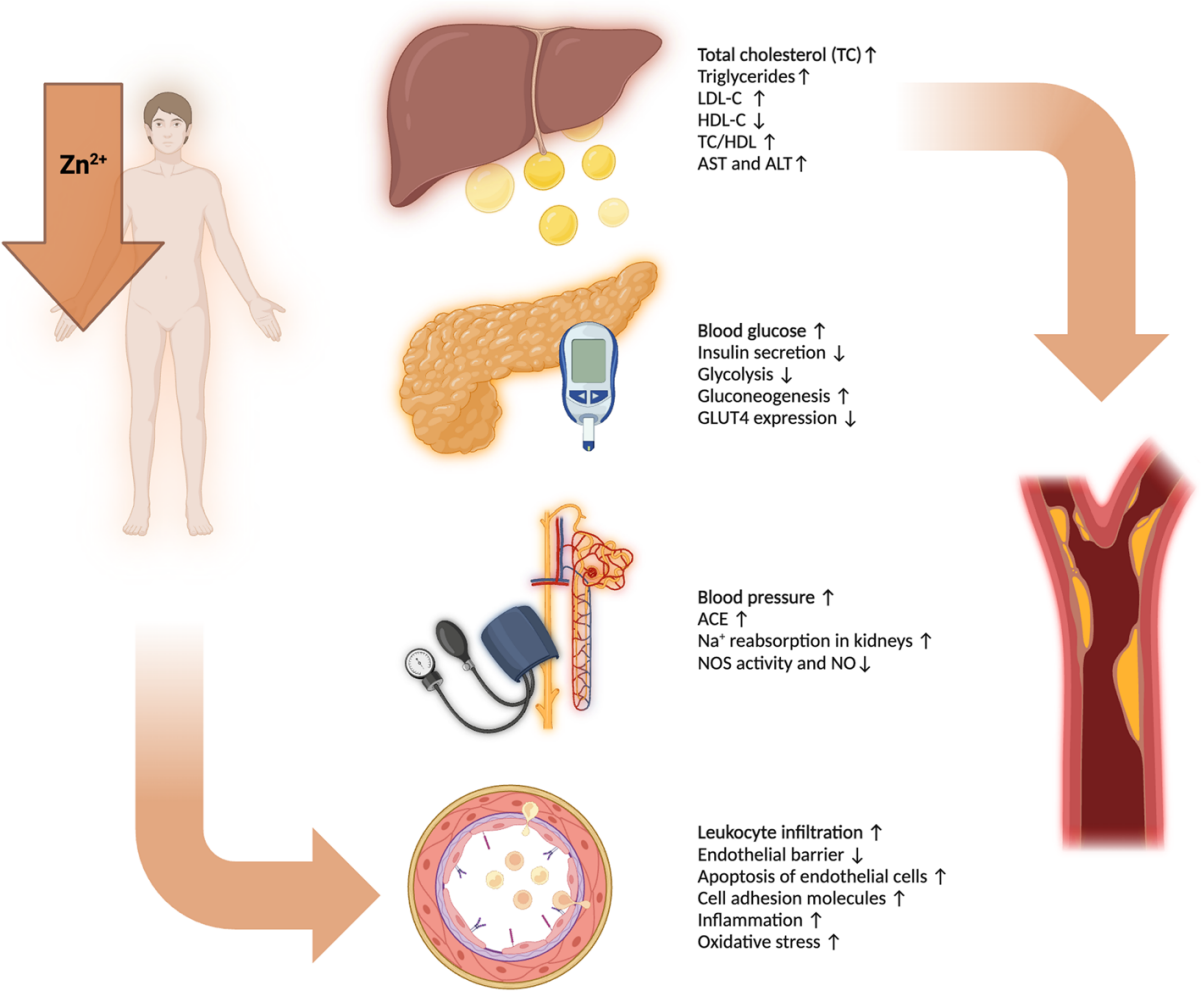
Impact of Zinc Deficiency on Atherosclerosis and Cardiovascular Health Zinc deficiency contributes to dysregulated lipid metabolism, glucose metabolism, elevated blood pressure and endothelial dysfunction. Together, these effects heighten the risk of atherosclerosis and metabolic disorders in zinc-deficient individuals. (↑): Characteristic increased, (↓): Characteristic decreased Created in https://BioRender.com

In sum, zinc is a vital component in both preventive and therapeutic strategies for AMD, especially for aging populations who are at increased risk. Through its multifaceted effects, such as antioxidant properties, inflammatory modulation, genetic interactions, and RPE function support, zinc supplementation presents a low-cost, accessible option to slow AMD progression and help maintain vision quality in elderly individuals Figure 7.

### Atherosclerosis

Atherosclerosis it a pathological condition characterized by the buildup of plaque, composed of fats, cholesterol, calcium and other substances, within the intima of large and medium-sized arteries- This process begins with the activation of endothelial cells, setting off a cascade that leads to vessel narrowing, activation of inflammatory pathways, and ultimately, formation of atheromatous plaques [443]. Atherosclerosis serves as the primary pathological mechanism behind most cardiovascular disease (CVD) cases, a leading global health concern and one of the top causes of disability and premature death [444, 445].

Importantly, atherosclerosis can begin early in life and often progresses silently over decades before symptoms emerge [446]. The prevalence of CVD rises sharply with age, affecting approximately 38% of individuals between 40 and 60 and reaching 83% in those over 85. Increased age is associated with conditions like elevated carotid intima-media thickness, carotid plaque, and carotid stenosis, which occur more frequently in older adults, compounding their CVD risk [447]. Given the rising prevalence of AS in elderly populations, the burden it places on personal health and healthcare systems presents a significant public health challenge requiring immediate attention.

Emerging evidence points to the critical role of zinc in modifying multiple risk factors associated with AS, particularly in older adults. Zinc impacts lipid metabolism, glucose metabolism, and blood pressure regulation, making it a promising therapeutic agent against AS (Fig. 7). Zinc-α2-glycoprotein (ZAG) is a glycoprotein with a zinc-binding site that plays a pivotal role in lipid metabolism. It promotes lipolysis through the cAMP signaling pathway [448] and enhances energy metabolism in brown adipose tissue by stimulating PPARγ and early B cell factor 2 (EBF2) [449]. Serum ZAG concentration declines progressively with age, showing an inverse correlation with fasting blood glucose and triglyceride levels, while higher body mass index (BMI) is also negatively associated with ZAG levels [450]. Furthermore, serum zinc concentrations are significantly lower in obese patients, with obesity being a major risk factor for CVD [451]. Zinc has been shown to correct lipid abnormalities in higher-fat diet models, lowering liver enzymes, including aspartate aminotransferase (AST) and alanine transferase (ALT), associated with liver stress [452]. The beneficial effects of zinc on lipid metabolism extend to improved cardiovascular outcomes. Zinc supplementation has been shown to significantly reduce total cholesterol (TC), increase high-density lipoprotein cholesterol (HDL-C) in individuals with underlying health conditions, and lower low-density lipoprotein cholesterol (LDL-C) and triglyceride levels, as demonstrated in a meta-analysis [453]. Additionally, studies assessing the TC to high-density lipoprotein cholesterol (TC/HDL-C) ratio, a recognized marker of CVD risk, indicate that zinc supplementation reduces this ratio, further highlighting its protective role in cardiovascular health [454].

Zinc also plays a protective role against atherosclerosis through its impact on glucose metabolism. Supplementation with zinc has been shown to reduce fasting glucose levels and HbA1c [455]. Additional effects of zinc on glucose metabolism will be explored in the diabetes mellitus section of this review.

Studies have demonstrated a negative association between serum zinc levels and high blood pressure [456]. Moreover, zinc supplementation has been shown to reduce systolic blood pressure, highlighting its potential therapeutic role in managing hypertension [457]. One critical organ for blood pressure regulation is the kidney, which is highly sensitive to zinc deficiency. In zinc deficient rats, kidney functions are compromised due to structural abnormalities, including reduced number of nephrons and renal fibrosis, and functional impairments, such as reduced glomerular filtration rate and proteinuria [458]. Notably, zinc plays a protective role at the cellular level, as it has been shown to significantly inhibit apoptosis in ATP-depleted renal cells [459]. Zinc-deficient rats also exhibited elevated levels of angiotensin II, along with augmented expression of angiotensin-converting enzyme 1 (ACE1) and the angiotensin receptors AT1 and AT2 at both the mRNA and protein levels. These changes enhance the activity of the renin-angiotensin system (RAAS), contributing to vasoconstriction, sodium retention, and heightened blood pressure, thereby exacerbating the risk of hypertension [460]. Further compounding the issue, zinc deficiency has been shown to upregulate the renal Na-Cl cotransporter, which increases sodium retention and further elevates blood pressure [461].

Endothelial cells (ECs) are central to the pathogenesis of atherosclerosis. Endothelial cell dysfunction, a key event, is associated with changes in hemodynamic forces. Areas of low shear stress trigger proatherogenic responses such as increased permeability, production of ROS, activation of NF-κB, and upregulation of adhesion molecules that attract leukocytes [462].

Zinc plays a critical role in maintaining endothelial health by supporting nitric oxide synthesis through the activation of endothelial nitric oxide synthase (eNOS), which is essential for vascular relaxation and anti-atherogenic effects [463]. The enzymatic activity of eNOS relies on its dimeric configuration, which is essential for its proper function. This dimeric state is stabilized by a zinc ion, playing a crucial role in maintaining the structural integrity and interaction of the two monomers [464]. The removal of zinc causes eNOS dimers to dissociate into inactive monomers in endothelial cells [465]. Hence, zinc supplementation increases intracellular NO production [466].

Moreover, Zinc deficiency enhances NF-κB activation, fueling inflammatory responses [467]. This pro-inflammatory state is further exacerbated by the reduced expression of PPARα and PPARγ in zinc-deficient endothelial cells, as these receptors are crucial inhibitors of NF-κB signaling, underlining zinc's vital role in preventing sustained inflammation [19]. Supplementation with zinc markedly reduces the binding activity of transcription factors NF-κB and AP-1, which in turn results in a significant decrease in the production of the pro-inflammatory cytokine IL-8 in endothelial cells [468]. Additionally, the hypoxia-inducible factor-1 (HIF-1) acts as a pivotal regulator during hypoxic and ischemic conditions, playing a key role in promoting cell survival and driving the production of pro-inflammatory cytokines. These actions contribute to critical processes such as angiogenesis and vascular remodeling [469]. Zinc has been shown to inhibit HIF-1α, impair its heterodimer formation and enhance its degradation via the proteasomal pathway [470].

Furthermore, a deficiency in zinc markedly impairs the barrier function of porcine endothelial cells [471], making them more prone to leukocyte infiltration and facilitating inflammatory processes [472]. Low zinc levels heighten endothelial cell vulnerability to LPS-induced apoptosis, whereas higher intracellular zinc supports defenses against apoptosis, possibly via nitric oxide pathways [473]. In addition, zinc-deficient endothelial cells exhibit increased caspase-3 activation, leading to higher rates of cell death, an effect reversible by zinc supplementation [474].

In atherosclerosis, endothelial cells produce chemokines, cytokines, and adhesion molecules that attract monocytes, leading to leukocyte migration into the vascular wall. Zinc deficiency exacerbates these processes by upregulating molecules like vascular cell adhesion molecule-1 (VCAM-1) and intercellular adhesion molecule-1 (ICAM-1), facilitating monocyte attachment and intensifying vascular inflammation and AS progression [475, 476].

Human studies support these findings: A randomized controlled trial demonstrated that a six-month zinc supplementation regimen in older adults increased plasma zinc levels and decreased markers of inflammation, including high-sensitivity C-reactive protein, IL-6, MCP-1, and VCAM-1. These findings suggest that addressing zinc deficiency in older populations may alleviate systemic inflammation and oxidative stress, thereby reducing the risk of atherosclerosis and improving cardiovascular outcomes [477].

Epidemiological studies have identified an inverse relationship between serum zinc levels and the risk of coronary artery disease (CAD) [478]. A meta-analysis further demonstrates that patients with heart failure have significantly lower serum zinc levels compared to healthy controls, with individuals diagnosed with idiopathic dilated cardiomyopathy exhibiting particularly reduced zinc concentrations [479]. Additionally, a significant inverse correlation has been observed between serum zinc levels and left ventricular mass, a marker of left ventricular hypertrophy [480]. Healthy individuals show higher serum zinc levels than those diagnosed with atherosclerosis or unstable angina [481, 482]. Furthermore, increased dietary zinc intake has been linked to a lower risk of coronary artery calcium progression, a known marker of subclinical atherosclerosis, particularly when the zinc is sourced from non-red meat [483]. Conversely, zinc intake from red meat sources has been positively associated with a higher risk of CVD [484]. Lastly, individuals who have suffered myocardial infarction were found to have significantly reduced zinc levels in both serum and hair samples [485].

This leads to the conclusion that zinc´s essential role in endothelial function, its modulation of inflammatory and oxidative stress pathway, and its impact on atherosclerosis progression position it as a vital nutrient for cardiovascular health in aging populations.

### Depression

Depression is one of the most prevalent mental health disorders worldwide, affecting approximately 280 million people [486]. Major Depressive Disorder (MDD) specifically has an alarming presence across age groups, with a 12-month prevalence of 10.4% and a lifetime prevalence of 20.6% [487]. MDD ranks among the top five disabling disorders globally [488], leading not only to mental health challenges but also to serious physical health risks, such as cardiovascular disease, stroke, and metabolic disorders [489, 490]. For aging populations, MDD presents an even greater concern, as it significantly raises the risk of Alzheimer´s disease, dementia, frailty, and reduced health span, underlining its multifaceted burden on both mental and physical health in older adults [491–493]. Around 22.1% of older adults with mild dementia and 11.6% of those with moderate dementia also experience major depressive disorder [494].

The implications of MDD on mortality are profound, with affected individuals facing increased risks of death from all causes, particularly cardiovascular mortality [489, 495]. Moreover, MDD has been linked to characteristics commonly associated with aging, suggesting that it may contribute to a premature aging phenotype, particularly in older adults who are already at heightened risk [489, 495].

Treatment for depression is typically stratified by severity, with mild depression managed through psychoeducation, self-care, and psychotherapy, while moderate to severe depression often requires a combination of psychotherapy and antidepressant medications [496]. Antidepressants remain a cornerstone of treatment, demonstrating clear benefits in reducing depressive symptoms compared to placebo [497]. However, the rising prevalence of depression in the general population is coupled with an increasing number of patients who do not respond to antidepressant therapy, underlining the need to identify underlying factors to enable early detection and intervention, especially in high-risk groups [498].

Research shows that low dietary zinc intake is linked to an elevated risk of depression [23, 499, 500]. Patients with major depression frequently exhibit lower serum zinc levels than healthy controls [501, 502] and a positive correlation exists between zinc deficiency and depression severity as these studies show among female students, postmenopausal women, and hemodialysis patients [503–505]. Studies with human subjects have shown that dietary or supplemental zinc interventions have antidepressant-like effects and improve mood [506–508].

Depression has been partly linked to inflammation, as evidenced by the role of proinflammatory cytokines like IL-1β in its development [509]. Zinc deficiency promotes the production of IL-1β by increasing the accessibility of the IL-1β promoter, which possibly results in a constitutive IL-1β production even in the absence of danger signals. Zinc repletion reduces promoter accessibility, thereby limiting IL-1β production under basal conditions, although it can still be induced by stimuli (danger signals) such as LPS [510]. This mechanism may contribute to an elevated risk of depression.

Furthermore, patients with major depression show a reduced number of Tregs in their peripheral blood, a characteristic also associated with inflammatory diseases [511]. Both antidepressant therapy [512] and zinc supplementation [327] have been found to reduce IL-1β expression and increase Treg levels. Similarly, both treatments lower the production of IFN-γ, further emphasizing their shared anti-inflammatory effects [327, 513].

Beyond its role in inflammation-related mechanisms contributing to depression, zinc also influences depression through other pathways. The G protein-coupled receptor 39 (GPR39) is responsive to zinc levels, and its activation influences pathways involved in depression [26, 514]. Zinc-deficient animals have been shown to exhibit reduced GPR39 expression in brain areas such as the hippocampus and frontal cortex, similar to observations in humans with depressive symptoms who died by suicide [515]. Mice lacking GPR39 display behaviors, seen in depression and anxiety, accompanied by compromised muscle coordination. Furthermore, these mice exhibited an altered expression of the Cl^−^ transporter KCC2 in the amygdala, which influences GABAergic signal pathways [516].

The influence of zinc on depression is further underlined by its role in regulating zinc transporter proteins. Studies of stress-induced depression models have revealed lower total zinc levels, accompanied by reduced expression of ZnT1 in the prefrontal cortex and hippocampus, as well as ZnT3 in the hippocampus [517, 518].

Depression-like behaviors resulting from zinc deficiency are associated with heightened hippocampal expression of the N-methyl-D-aspartate receptor (NMDAR), and lowered α-amino-3-hydroxy-5-methyl-4-isoxazolepropionic acid receptor (AMPAR) expression. Effects that can partially be reversed with fluoxetine treatment [519, 520].

These findings underline the importance of dietary zinc intake, particularly for aging populations who are at higher risk of both zinc deficiency and depression-related cognitive decline. Evidence from human studies suggests that dietary or supplemental zinc can have antidepressant effects, providing a promising adjunctive treatment approach for individuals who do not fully respond to conventional antidepressant therapy [506–508]. Recognizing zinc´s potential to support mental health, particularly in older adults, can guide treatment strategies aimed at improving both psychological and physiological outcomes in populations vulnerable to accelerated aging and mental health challenges associated with depression.

### Wound healing

Elderly individuals are more susceptible to chronic wound, such as diabetic and pressure ulcers, often exacerbated by age-related declines in wound healing linked to aging immune system [521, 522]. Research highlights that essential reparative functions, including angiogenesis, fibroblast activity, and collagen deposition, are compromised in older adults. This delayed wound closure not only increases infection risks but also extends hospital stays, imposing added strain on healthcare resources [523, 524]. As wound healing is essential to tissue integrity and overall health, the care and management of chronic wounds in aging populations have become a critical public health concern, annual cost of treating chronic wounds, including various ulcers, are estimated to reach up to $96 billion in the U.S. alone [525].

The skin holds a relatively high concentration of zinc, which constitutes about 6% of the body´s total zinc content and is mainly concentrated in the skin´s epidermis. This zinc is especially significant in the wound healing process [526]. During the wound healing process, initial hemostasis is established through the aggregation of platelets and the formation of a clot, which is subsequently stabilized by the secretion and polymerization of fibrin [527]. Studies involving both rats and humans demonstrated that dietary zinc deficiency leads to impaired platelet aggregation [528, 529]. Zinc plays a crucial role in facilitating platelet aggregation by interacting with the fibrinogen receptor glycoprotein IIb/IIIa complex, enhancing the response synergistically when combined with collagen or ADP [530, 531].

In the inflammatory phase, a critical step in wound healing, zinc levels increase significantly. Rat model studies demonstrate rise of about 15% in zinc within 24 h post-injury at the wound margins, followed by an increase of up to 30% during the formation of granulation tissue and epidermal proliferation [532, 533]. This zinc signal initiates the repair process in epithelial cells [534].

Once inflammation subsides, re-epithelialization begins, typically around 2–3 days after injury, as fibroblasts infiltrate the wound site, initiating collagen and extracellular matrix (ECM) deposition to create a scaffold for tissue repair. This matrix serves a foundation supporting the migration of epithelial cells, keratinocytes, and microvascular structures [535]. The TGF-β/SMAD pathway, which zinc supports as an essential cofactor, regulates ECM deposition, aiding granulation tissue formation [325]. Additionally, zinc-dependent matrix metalloproteinases (MMPs) are involved in depredating granulation tissue to enable migration of keratinocytes, fibroblasts, and endothelial cells, promoting cell growth, migration, and angiogenesis [536]. Both zinc and calcium are essential for the efficient functioning of MMPs in vitro [537, 538].

Clinical evidence suggests that zinc deficiency can impair wound healing as low serum zinc levels correlate with poor healing outcomes, including infection and dehiscence in patients undergoing hip replacement surgeries [22]. Chronic leg ulcer patients commonly present with zinc metabolism irregularities, leading to low serum zinc levels [539–541]. Studies have shown that topical zinc application can enhance wound repair, as demonstrated in porcine skin wound models where zinc accelerated re-epithelization [542] and increased keratinocyte migration, contributing to epidermal re-epithelization [543]. Zinc oxide, specifically, has been effective in ulcer healing, performing comparably to enzymatic debriding agents for pressure ulcers [544–546] and demonstrating effectiveness in diabetic foot ulcers by promoting debridement through zinc oxide-medicated occlusive dressings, outperforming standard hydrocolloid dressings that utilize autodebridement [547]. In a study comparing topical zinc treatments with saline controls in patients with uncomplicated wounds, those treated with zinc showed significantly better healing outcomes [548].

Zinc oxide also aids in collagen degradation in neurotic wounds, which is beneficial in cases of tissue damage requiring necrotic tissue breakdown for effective healing [538]. Nutritional interventions have also shown benefits. For example, a high-calorie, high-protein oral formula fortified with zinc and other nutrients led to significant ulcer reduction in malnourished patients with chronic pressure ulcers, although zinc´s exact role within the formula remains uncertain [549]. In surgical settings, zinc infusions administered pre- and post-operatively in vascular surgery patients prevented the typical postoperative drop in serum zinc levels, resulting in a reduction in wound healing complications compared to the placebo group [550].

However, studies on systemic zinc supplementation present mixed results. For instance, a systemic review indicated that zinc sulfate did not significantly aid in treating leg ulcers unless zinc deficiency was evident [551]. In contrast, another study found general poor dietary zinc intake among patients with leg ulcers [552]. Topical zinc therapy, however showed improved healing in chronic venous leg ulcers in a meta-analysis [553].

**Fig. 8:**
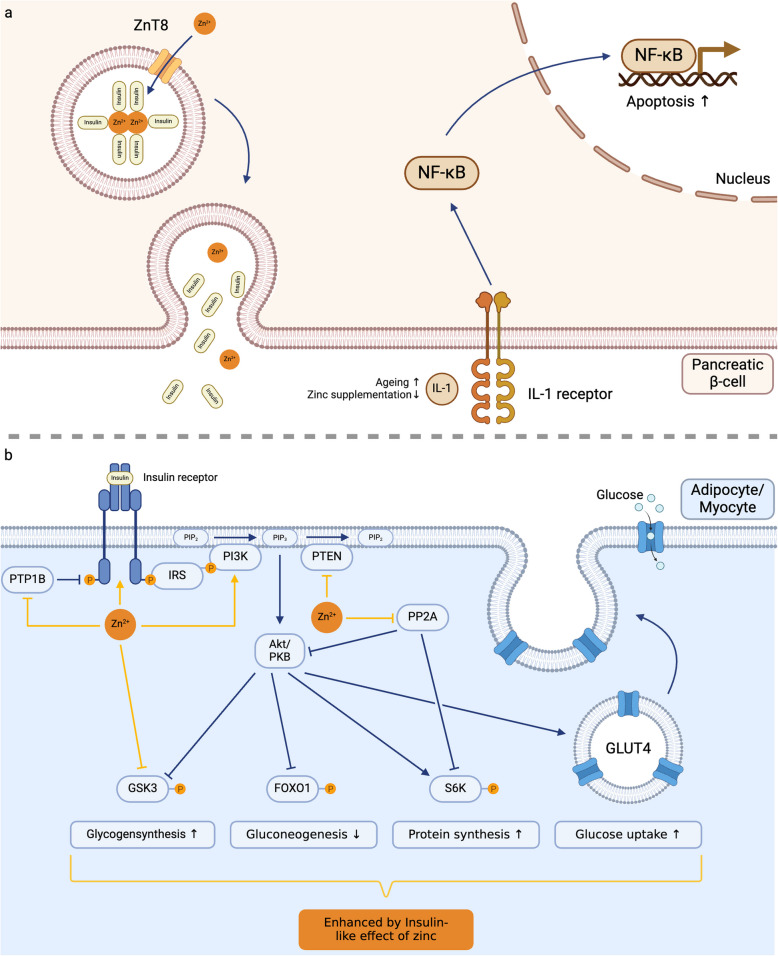
Impact of Zinc on Insulin Secretion, β-Cell Health, and Insulin Signaling (↑): increased, (↓): decreased, () activation, (–I): inhibition (**a**) Zinc transporter 8 (ZnT8) facilitates zinc storage in insulin granules within pancreatic β-cells, playing a critical role in insulin storage. Aging and zinc deficiency increase IL-1, exacerbating IL-1-mediated activation of the NF-κB pathway, which leads to increased apoptosis β-cells. **b** Zinc plays a modulatory role in insulin signaling pathways in target tissues such as adipocytes and myocytes. Zinc inhibits protein tyrosine phosphatase 1B (PTP1B) activity, enhancing insulin receptor signaling through IRS and PI3 K pathways. This supports Akt/PKB activation, promoting downstream effects such as increased glucose uptake via GLUT4 translocation, enhanced glycogen synthesis (via inhibition of GSK3), reduced gluconeogenesis (via FOXO1 suppression), and increased protein synthesis (via S6 K activation). Created in https://BioRender.com

In summary, zinc plays a multifaceted role in wound healing, making it a critical component of wound care for elderly individuals. From enhancing hemostasis and inflammation to promoting re-epithelization and ECM remodeling, zinc´s physiological functions support each phase of the wound healing process. For the aging population, which is at higher risk of delayed wound healing and associated complications, zinc-based treatments, particularly topical applications, offer promising therapeutic options, though the effectiveness of systemic supplementation remains context-dependent Figure 8.

### Diabetes mellitus

Diabetes mellitus is a chronic metabolic disorder marked by elevated blood glucose levels, or hyperglycemia, and increased glycated hemoglobin, sometimes accompanied by glycosuria [554, 555]. This condition results from disruption in glucose metabolism, often stemming from insufficient insulin production by the pancreas, reduced in insulin effectiveness in body tissues (insulin resistance), or a combination of these factors, which is particularly common among older adults [556]. Prolonged hyperglycemia can lead to progressive complications, damaging vital organs such as the heart, blood vessels, eyes, kidneys, and nerves, underscoring the need for targeted management of diabetes in older populations to prevent these outcomes [556].

Diabetes ranks as the fifth leading cause of death globally, responsible for approximately 1.6 million fatalities per year [557, 558], and accounted for 11.3% of all global deaths in 2019 [559]. Type 2 diabetes, in particular, has seen a continuous increase in prevalence, driven by lifestyle shifts and population aging, especially in Westernized societies, where these factors contribute to rising obesity rates. Among those aged 70 and above, the prevalence of diabetes is notably high, affecting around 25–30% of this demographic group [560, 561].

In pancreatic β-cells, the highest concentration of zinc is in insulin secretory granules, where it plays a vital role in insulin stability and secretion. Insulin binds with zinc ions to form hexamers, which allow dense packing of insulin in storage vesicles, protecting it from degradation [50, 562]. The zinc transporter ZnT8 is essential for moving zinc into pancreatic β-cell granules, facilitating zinc-insulin hexamer formation, critical for efficient insulin release. Zinc deficiency can reduce ZnT8 expression, thereby hindering insulin secretion [563–565]. Single nucleotide polymorphisms (SNPs) as the rs13266634 SNP in the ZnT8 gene and is associated with an increased risk of developing type 2 diabetes [566] The risk of developing type 2 diabetes with this SNP can be lowered with zinc supplementati[on [567].

Inflammaging plays a critical role in the pathogenesis of diabetes and the destruction of pancreatic β cells as well. A key contributor to this process is the pro-inflammatory cytokine IL-1β, which is significantly elevated in aging individuals [568]. IL-1β inhibits insulin release and processing of proinsulin into insulin, impairing glucose metabolism [569]. Furthermore, IL-1β induces apoptosis in pancreatic β cells through the activation of the NF-κB pathway, exacerbating β cell loss [570]. The potential to counteract these effects has been demonstrated with IL-1 receptor antagonists, which have been shown to improve glucose tolerance and enhance insulin production [571]. Zinc supplementation offers a promising way to mitigate these effects, as it has been shown to reduce symptoms of inflammaging and lower IL-1β release. Ex vivo studies reveal that zinc supplementation can decrease IL-1β output following LPS stimulation [572, 573]. These findings suggest that zinc may help reducing the risk of diabetes mellitus in aging populations by protecting β cells from apoptosis.

The insulin signaling pathway, critical for glucose regulation, begins with insulin binding to its receptor, which activates insulin receptor substrates (IRS) and recruits phosphoinositide 3-kinase (PI3 K), which turns phosphatidylinositol-4,5-bisphosphate (PIP_2_) into phosphatidylinositol-3,4,5-trisphosphat (PIP_3_). PIP_3_ then activates Akt (Protein Kinase B), initiating various metabolic processes: GLUT4 translocates to the cell membrane to facilitate glucose uptake, glycogen synthase kinase 3 (GSK3) is inhibited to promote glycogen synthesis, and FOXO1 is phosphorylated, which reduces gluconeogenesis. Additionally, Akt activates ribosomal protein S6 K, enhancing protein synthesis and supporting cellular growth and metabolism. Phosphatases, such as PTEN and PTP1B, regulate this pathway by deactivating signals, thereby maintaining balance in glucose and metabolic activity [574].

Zinc is a crucial element in activating the PI3 K/Akt pathway (Fig. 8), which is vital for effective insulin signaling [575]. Research indicates that zinc independently stimulates the activation of insulin receptor β, AKT, ERK1/2 and Src homology-2 domain-containing protein tyrosine phosphatase (SHP) in myotubes, promoting glucose uptake via GLUT4 translocation in the cell membrane [575, 576]. Inhibition of the insulin receptor in C2 C12 skeletal muscle cells reduces zinc-induced Akt activation, suggesting that zinc may exert its effects through insulin receptor interaction [575], more exactly by enhancing the phosphorylation of the insulin receptor´s β-subunit [577, 578].

Zinc is also inhibits the enzyme tyrosine phosphatase 1B (PTP1B), which would otherwise reduce the insulin signal by dephosphorylating the β-subunit of the insulin receptor [579]. Zinc also inhibits the phosphatase and tensin homolog (PTEN), which normally dephosphorylates PIP_3_, thereby suppressing the activation of Akt. Through PTEN inhibition, zinc promotes the further activation of Akt signaling [580, 581]. This elevated insulin signaling through zinc causes more GLUT4 transporters to move to the cell membrane, increasing glucose uptake into cells [576]. It also enhances the phosphorylation of forkhead box protein O1 (FOXO1), which suppresses gluconeogenesis [582]. Additionally, it activates S6 K, boosting protein synthesis [583], and inhibits GSK3 through phosphorylation, leading to greater glycogen synthesis [584]. GSK3 is also directly inhibited by zinc [585]. Furthermore, zinc inhibits PP2 A, which otherwise would inhibit Akt and S6 K [230, 586].

Diabetic individuals, especially older adults, often exhibit lower serum zinc levels than healthy controls. Low zinc levels are associated with higher fasting blood sugar and HbA1c levels, indicating poorer glycemic control in type 2 diabetes mellitus [587–589]. Zinc deficiency correlates with the severity of diabetic complications, including peripheral neuropathy. In case–control study, diabetic patients with peripheral neuropathy had lower serum zinc levels, and zinc levels were negatively correlated with HbA1c, neuropathy scores (NSC and MNSI), and nerve conduction [590]. Zinc supplementation has shown promising effects in improving neuropathic symptoms. Patients with neuropathy treated with zinc sulfate showed significant improvements in fasting and postprandial blood glucose and motor nerve conduction velocity compared to the placebo group [591, 592]. Additionally, zinc supplementation may reduce the risk of diabetic nephropathy complications [593].

Studies also indicate that zinc supplementation in type 2 diabetes mellitus patients can reduce fasting blood glucose, HbA1c, and lipid profiles, including TC and LDL-C, while increasing HDL-C, highlighting its potential as a beneficial adjunct therapy for older adults with diabetes [455, 594, 595].

Given the significant prevalence of diabetes among older adults, zinc supplementation offers an accessible strategy to improve glycemic control, mitigate complications, and enhance overall health and quality of life in this vulnerable population.

## Conclusion

In conclusion, zinc is a crucial nutrient that supports immune health and reduces inflammation in older adults, providing valuable protection against age-related diseases. Zinc plays multiple roles in the immune system by strengthening the activity of various immune cells, including granulocytes, NK cells, macrophages, T cells, and B cells. As we age, immune function often weakens, leading to a higher risk of infections, chronic low-level inflammation, and reduced cellular function, challenges that zinc can help address through targeted support of these immune cells.

Zinc supports healthy cell function, which helps lower the risk of cancer and infections, reduces age-related inflammation/inflammaging, and boosts Treg activity, thereby decreasing the likelihood of autoimmune diseases. Zinc’s role in reducing oxidative stress and modulating inflammatory pathways further suggests its potential to delay or reduce the progression of specific age-related diseases, including age-related macular degeneration, diabetes, depression, atherosclerosis, and impaired wound healing. In the case of AMD, zinc is integral in combating oxidative damage in retinal cells, thereby slowing the disease’s progression and preserving vision. For diabetes, zinc’s involvement in glucose metabolism and insulin regulation can help reduce inflammatory markers associated with the disease, potentially aiding in glycemic control and protecting against diabetes-related complications. In the context of depression, zinc's anti-inflammatory and neuroprotective effects show promise in managing age-related mental health challenges by reducing inflammation that may contribute to mood disorders. Atherosclerosis, a condition driven by chronic inflammation in blood vessels, may also be mitigated by zinc’s anti-inflammatory actions, supporting cardiovascular health by reducing the buildup of plaques in arterial walls. Additionally, zinc plays a role in collagen synthesis and cellular repair, which is critical in improving wound healing, a process that often becomes compromised with age.

This review underlines the essential nature of maintaining adequate zinc levels in promoting immune health, resilience, and reducing the risk of chronic conditions in older adults. Considering its low cost, safety, and accessibility, zinc presents itself as a valuable intervention to support healthy aging and improve quality of life. By targeting both cellular health and inflammation, zinc addresses multiple facets of immunosenescence, making it a key component in comprehensive strategies to enhance immunity, delay age-related diseases, and bolster overall well-being in elderly populations.

## Data Availability

No datasets were generated or analysed during the current study.

## Abbreviations

AID: Activation-induced cytidine deaminase
Akt: Protein Kinase B
ALI: Acute lung injury
AMD: Age-related macular degeneration
AP-1: Activator protein-1
APC: Antigen presenting cell
BCR: B cell receptor
Breg: Regulatory B cell
CFH: Complement factor H
CVD: Cardiovascular disease
DC: Dendritic cell
DGE: German Nutrition Society
ECM: Extracellular matrix
EFSA: European Food Safety Authority
eNOS: Endothelial nitric oxide synthase
ERK1/2: Extracellular-signal regulated kinases 1/2
fMLP: N-formylmethionyl-leucyl-phenylalanine
FOXO1: Forkhead box protein O1
G-CSF: Granulocyte-colony stimulating factor
GM-CSF: Granulocyte–macrophage colony stimulating factor
GPR39: G protein-coupled receptor 39
GSK3: Glycogen synthase kinase 3
HCC: Hepatocellular carcinoma
HDL-C: High-density lipoprotein cholesterol
HIF-1: Hypoxia-inducible factor-1
HLA: Human leucocyte antigen
HSC: Hematopoietic stem cell
IFN: Interferon
Ig: Immunoglobulin
IL: Interleukin
iNOS: Inducible nitric oxide synthase
IRS: Insulin receptor substrates
KIR: Killer cell immunoglobulin-like receptor
LDL-C: Low-density lipoprotein cholesterol
LPS: Lipopolysaccharide
MAPK: Mitogen-activated protein kinase
MCP-1: Monocyte chemotactic protein-1
mDC: Myeloid dendritic cell
MDD: Major Depressive Disorder
MHC: Major histocompatibility complex
MLC: Mixed lymphocyte culture
MMP: Matrix metalloproteinase
MPO: Myeloperoxidase
MT: Metallothionein
mTORC1: Mammalian target of rapamycin complex 1
NET: Neutrophils release extracellular trap
NF-κB: Nuclear factor kappa-light-chain-enhancer of activated B-cells
NIH: National Institutes of Health
NK cells: Natural killer cell
NLR: Neutrophil-to-lymphocyte ratio
PAMP: Pathogen-associated molecular patterns
PBMC: Peripheral blood mononuclear cell
PD-1: Programmed Cell Death Protein 1
PD-L: Programmed Death-Ligand
pDC: Plasmacytoid dendritic cell
PHA: Phytohemagglutinin
PI3 K: Phosphoinositide 3-kinase
PIP_3_: Phosphatidylinositol-3,4,5-trisphosphat
PKC: Protein kinase C
PMN: Polymorphonuclear neutrophil
PP2 A: Protein phosphatase 2
PPAR: Peroxisome proliferator-activated receptor
PTEN: Phosphatase and tensin homolog
PTP1B: Tyrosine phosphatase 1B
ROS: Reactive oxygen species
RPE: Retinal pigment epithelium
S6 K: S6 kinase
SNP: Single nucleotide polymorphism
SOD: Superoxide dismutase
TC: Total cholesterol
TCR: T cell receptor
TGF: Transforming growth factor
TLR: Toll-like receptor
TNF: Tumor necrosis factor
TPEN: *N*,*N*,*N′*,*N′*-Tetrakis(2-pyridinylmethyl)-1,2-ethanediamine
Tregs: Regulatory T cell
VCAM-1: Vascular cell adhesion molecule-1
VEGF: Vascular endothelial growth factor
WHO: World Health Organization
XCI: X chromosome inactivation
Xi: Inactive X chromosome
ZAG: Zinc-α2-glycoprotein
ZD: Zinc deficiency
ZnT: Zinc transporter
